# Mesenchymal ovarian cancer cells promote CD8^+^ T cell exhaustion through the LGALS3-LAG3 axis

**DOI:** 10.1038/s41540-023-00322-4

**Published:** 2023-12-12

**Authors:** Edward Yakubovich, David P. Cook, Galaxia M. Rodriguez, Barbara C. Vanderhyden

**Affiliations:** 1 Department of Cellular and Molecular Medicine, University of Ottawa, Ottawa, ON Canada; 2 Cancer Therapeutics Program, Ottawa Hospital Research Institute, Ottawa, ON Canada; 3 Center for Infection, Immunity and Inflammation, University of Ottawa, Ottawa, ON Canada

**Keywords:** Computational biology and bioinformatics, Cancer, Immunology, Regulatory networks

## Abstract

Cancer cells often metastasize by undergoing an epithelial-mesenchymal transition (EMT). Although abundance of CD8^+^ T-cells in the tumor microenvironment correlates with improved survival, mesenchymal cancer cells acquire greater resistance to antitumor immunity in some cancers. We hypothesized the EMT modulates the immune response to ovarian cancer. Here we show that cancer cells from infiltrated/inflamed tumors possess more mesenchymal cells, than excluded and desert tumors. We also noted high expression of *LGALS3* is associated with EMT in vivo, a finding validated with in vitro EMT models. Dissecting the cellular communications among populations in the tumor revealed that mesenchymal cancer cells in infiltrated tumors communicate through *LGALS3* to *LAG3* receptor expressed by CD8^+^ T cells. We found CD8^+^ T cells express high levels of *LAG3*, a marker of T cell exhaustion. The results indicate that EMT in ovarian cancer cells promotes interactions between cancer cells and T cells through the *LGALS3* - *LAG3* axis, which could increase T cell exhaustion in infiltrated tumors, dampening antitumor immunity.

## Introduction

The epithelial-to-mesenchymal transition (EMT) refers to the plastic ability of epithelial cells to undergo a conversion to a mesenchymal state by gradually shedding epithelial features in response to environmental signals^1^. In tissue microenvironments, cells undergoing EMT exist on a continuum where cells are either in a complete epithelial state, complete mesenchymal cells, or a gradient of intermediate states. In cancer, EMT has been associated with metastatic spread and immunosuppression^2–4^. In the case of metastasis, cancer cells responsible for invasiveness and migration appear to be at least in a partial EMT (pEMT), if not a fully mesenchymal, state^5–7^. Mesenchymal and pEMT states also highly contribute to the immunosuppressive burden in the ovarian tumor microenvironment (TME)^7–9^.

Immune infiltration and inflammation play an important role in tumor development in a variety of cancers^10,11^. Chronic inflammation established by immune infiltration has been associated with tumor progression, particularly with enhanced metastasis and the EMT process through secreted factors such as transforming growth factor beta 1 (TGF-β1) and tumor necrosis alpha (TNF-α) derived from myeloid cells^12,13^. Human tumors can be classified according to their level of immune infiltration: an ‘infiltrated-inflamed’ (infiltrated) tumor is characterized by a TME containing tumor infiltrating lymphocytes (TILs) in intraepithelial locations and displaying a cytotoxic transcriptional signature^14^. This immune phenotype along with the presence of CD8^+^ cytotoxic T lymphocytes (CTLs) has been associated with better prognosis and overall survival in high-grade serous (HGSOC)^15,16^. ‘Immune-excluded’ (excluded) tumors are distinguished by TILs found mainly in the stromal portion of the tumor. In ‘immune-desert’ (desert) tumors, immune cells are largely absent from the TME^14,17^. The differences between excluded and desert tumors may be due to immune exclusion because of the density of the extracellular matrix^18^ or through a chemorepellent gradient as has been reported for triple-negative breast cancer^19^.

Even though in ovarian cancer, particularly in HGSOC, immune infiltration by CD8^+^CD103^+^ CTLs has been associated with increased progression-free survival, recent findings indicate that a large portion of this population are positive for T-cell immunoglobulin and mucin-domain containing-3 (TIM3)^20,21^ and Lymphocyte Activation Gene-3 (LAG3)^21,22^ in epithelial ovarian cancer (EOC), suggesting they are prone to exhaustion and attenuated effector functions. Both TIM3 and LAG3 act as co-inhibitory molecules together with programmed cell death protein-1 (PD1) to dampen CTL mediated antitumor immunity^22,23^. *LAG3* encodes a cell surface protein that is expressed on T-cells, natural killer (NK) cells, plasmacytoid dendritic cells (pDCs) and B cells, and associates with the T-Cell Receptor complex (TCR, CD3) on both CD4^+^ and CD8^+^ T cells acting as an immune inhibitory checkpoint. LAG3 binds to Galectin-3 (GAL-3) with strong affinity and chronic LAG3 engagement on CD8^+^ tumor antigen-specific T cells has been implicated in exhaustion of TILs and reduction in their cytolytic capacity^24,25^.

EMT in cancer cells has been linked to chemoresistance and immunosuppression in HGSOC. TGF-β/SMAD signaling drives resistance to paclitaxel, BMP9 activates EMT through TGF-β1 and promotes platinum resistance, and expression of lysyl oxidase (LOX) induces EMT through SLUG and TWIST1 and contributes to chemoresistance in activating PI3K/AKT^9,26^. Recent attempts in breast cancer to elucidate the role of EMT in cancer cells’ ability to facilitate an immunosuppressive TME have shown that mesenchymal cancer cells express low levels of MHC I and are associated with infiltration of regulatory T cells (T_reg_)^27^ and resistance to anti-CTLA4 therapy^3^. In HGSOC, lower abundance of CD8^+^ TILs is associated with the worst prognosis and a high expression of EMT-related gene signatures in cancer cells^28^. Despite the interest in how cancer cells affect CD8^+^ T-cell exhaustion in HGSOC, the role of the EMT has yet to be elucidated.

To better understand the signaling pathways underlying CD8^+^ T-cell function in HGSOC driven by EMT, we accessed a publicly available single-cell RNA-seq (scRNA-seq) dataset of 16 HGSOC samples^29^ categorized by their tumor immune phenotypes: infiltrated/inflamed (infiltrated), immune-excluded (excluded), or immune-desert (desert). We first discovered that infiltrated ovarian tumors have a higher EMT signature than either excluded or desert tumors. Pathway analysis revealed that EOC cancer cells in a pEMT or mesenchymal state upregulate pathways related to chemotaxis of immune cells, particularly in infiltrated TMEs. In-depth analysis of cell communication networks of cancer cells signaling to the CD8^+^ T cell population revealed that a large portion of the communication is facilitated by the mesenchymal cells and is mediated by HLA II molecules and LGALS3 communicating with the LAG3 receptor complex. Our findings suggest that direct signaling between mesenchymal cancer cells and CD8^+^ T cells trigger LAG3 and other T cell exhaustion pathways, disrupting anti-tumor immunity and supporting tumor development.

## Results

### Comparing single-cell profiles of distinct immune phenotypes of HGSOC

**Fig. 1:**
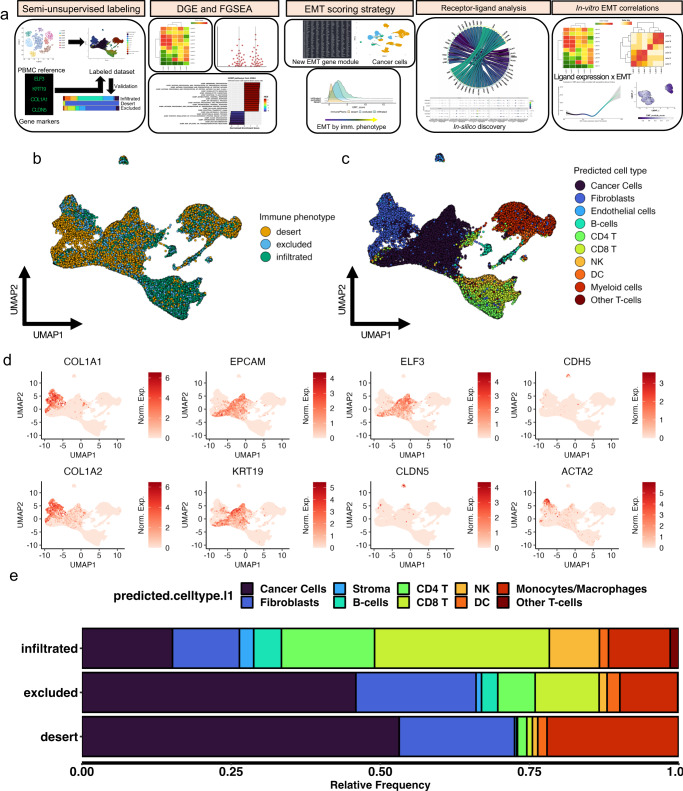
Identification of cell populations from patient-derived tumor cells in silico confirms greater immune cell presence in infiltrated tumors compared to excluded and desert tumors. **a** Overview of analysis and experimental validation procedures. **b** Uniform manifold and approximation projection (UMAP) of patient derived tumor cells colored by tumor immune phenotype. **c** Labeled UMAP from patient derived tumor cells colored by cell population. **d** Series of UMAPs colored by individual gene expression levels in various cell populations from patient derived tumors: *KRT19, ELF3, EPCAM* for cancer cells; *COL1A1*, *COL1A2* for fibroblasts; *CDH5, CLDN5* for endothelial cells; *ACTA2* for smooth muscle cells. **e** Relative frequency of overall cell populations in patient derived tumor samples separated by immune phenotype and colored by cell population.

To assess how EMT contributes to the activity, immunosuppression and/or exhaustion of CD8^+^ T cells, we began by accessing a scRNA-Seq dataset of 16 human ovarian cancers^29^. The plan for this data analysis is summarized in Fig. 1a. We chose these datasets due to their inclusion of immune phenotyping metadata confirmed by histology. We assessed for quality-control and clustered every sample individually by merging the respective CD45^+^, tumor, and stromal fractions of each individual dataset before using our semi-supervised labeling method to identify the individual cell types comprising each tumor sample, as described in the Methods. The tumor samples analyzed are from a single study where each tumor was processed with an identical protocol^29,30^. Although this protocol may introduce cell type biases, the assumption is that they are consistent among samples.

First, we overlaid the immune phenotypes of the samples in a UMAP to visualize the grouping of all the cells (Fig. 1b). Next, we overlaid the results from our semi-supervised labeling method (Fig. 1c, d) to label the cell types onto the UMAP. While there is some intracellular heterogeneity, most populations group together irrespective of the tumor immune phenotype. This suggests that infiltration status phenotypes minimally contributes to dimensionality reduction and clustering of the cell populations. By examining the population proportion breakdown in the TME by immune phenotype metadata (Fig. 1e, Supplemental Fig. 1), we found that semi-supervised labeling of the 16 tumors matches the originally published findings with respect to immune phenotype, where in infiltrated tumors there are most CD45^+^ cells relative to cancer cells, fewer CD45^+^ cells relative to cancer cells in excluded tumors, and fewest CD45^+^ cells relative to cancer cells in desert tumors. This confirms that our cell labeling method was accurate in correctly identifying CD45^+^ and CD45^-^ cells.

**Fig. 2:**
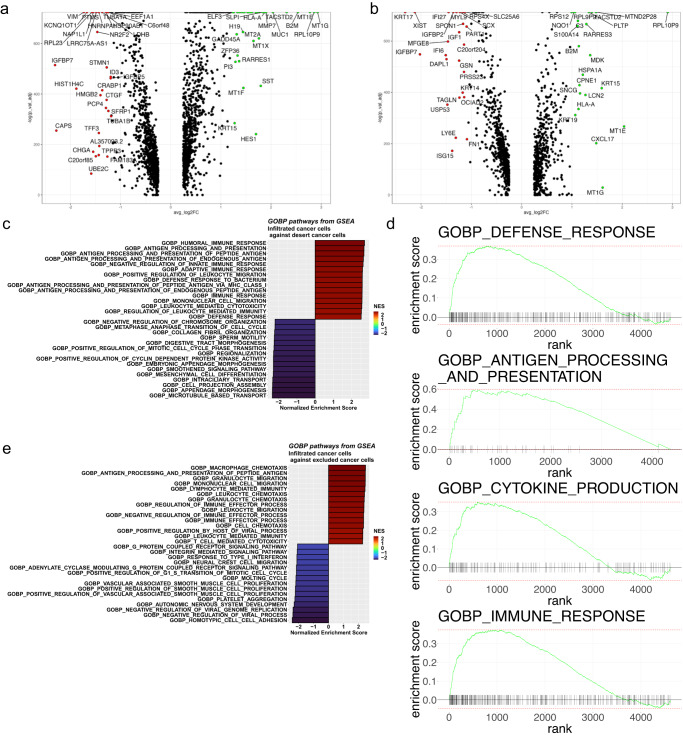
Cancer cells from infiltrated tumors upregulate signaling pathways related to immune cell chemotaxis, immune modulation, and antigen presentation compared to cancer cells from excluded or desert tumors. **a** Volcano plots of differentially expressed genes (DGE) between cancer cells of desert and infiltrated tumors (left) and excluded and infiltrated tumors (right). **b** Enrichment plot of infiltrated GOBP terms from GSEA of a selection of significant (*p-adjusted <0.05) pathways between cancer cells of infiltrated (red) and desert tumors (blue). **c** Enrichment plot of infiltrated GOBP terms from GSEA of a selection of significant (*p-adjusted <0.05) pathways between cancer cells of infiltrated (red) and excluded tumors (blue). **d** GSEA enrichment ranks of significantly upregulated (*p-adjusted <0.05) pathways related to immune regulation in cancer cells from infiltrated tumors. **e** Enrichment plot of infiltrated GOBP terms from GSEA of a selection of significant (*p-adjusted <0.05) pathways between cancer cells of infiltrated (red) and desert tumors (blue).

To assess the role of cancer cells in contributing to the unique composition of each immune phenotype, we performed differential gene expression analysis to identify the top and bottom most differentially regulated genes between cancer cells from the infiltrated TME compared to cancer cells from desert TME (Fig. 2a) and excluded TME (Fig. 2b). Cancer cells from infiltrated TMEs differentially express genes related to immune regulation within the context of carcinogenesis, such as: *B2M*^31^, *SLPI*^32^, *HLA-A*^33^, *ELF3*^34^, *MUC1*^35^, *MDK*^36^, *CXCL17*^37^ and others. Based on upregulation of these immune regulatory genes, we performed GSEA first comparing cancer cells from the infiltrated to the desert TMEs. We discovered that many biological pathways (GOBP) related to immune regulation were upregulated in cancer cells from infiltrated TMEs (Fig. 2c, d). Among the main findings, some pathways were related to chemotaxis and enrichment for processes related to antigen presentation and cytokine activity, suggesting that cancer cells in infiltrated tumors actively participate in modulating the immune response and shape the tumor immune phenotype. Similar results were obtained when investigating the pathway enrichment analyses comparing cancer cells from infiltrated versus excluded TMEs (Fig. 2e) and excluded to desert TMEs (Supplemental Fig. 2) suggesting that pathways related to immune regulation could be “turned on” either as a response to immune infiltration or as a precursor to the arrival of immune cells promoted by cancer cells themselves. These results may be indicative of EMT-related cell-cell signaling driving chemotaxis of various CD45^+^ cells from different populations to the TME.

### Cancer cells from infiltrated tumors are more mesenchymal compared to cancer cells from other immune phenotypes

**Fig. 3:**
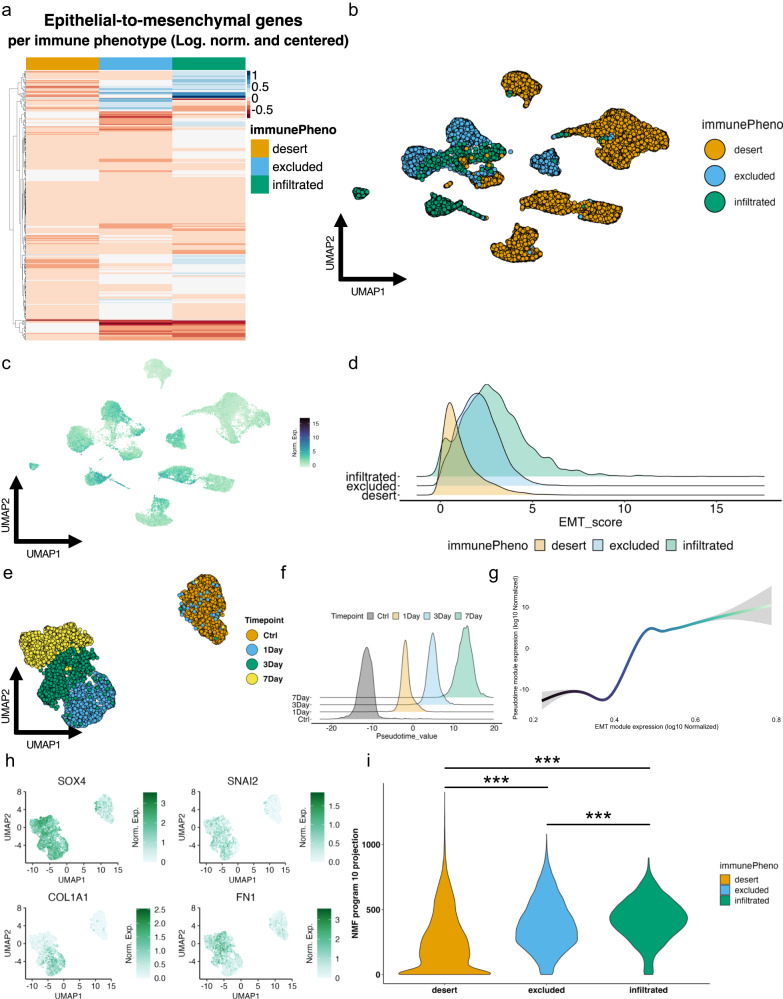
Cancer cells from infiltrated tumors have higher cancer-specific EMT signature scores compared to cancer cells from excluded and desert tumors and are more progressed on the EMT axis. **a** Quantile scaled heatmap of cancer-specific EMT signature genes arrayed by immune phenotype. **b** UMAP clustering of cancer cell compartment of scRNA-Seq dataset of 16 HGSOC tumors colored by the immune phenotype of the specific source tumor. **c** Enrichment UMAP plot for the cancer-specific EMT signature scores in the clustered cancer cell compartment. **d** Ridge plot of individual cancer-specific EMT signature module scores in the cancer cell compartment of each tumor’s immune phenotype. **e** UMAP clustering of OVCA420 cells treated with TGF-β1 for 4 different timepoints: Ctrl (no treatment), 1-day, 3-day, and 7-day. **f** Pseudotime value density for each individual treatment timepoint in OVCA420 cells treated with TGF-β1. **g** Generalized additive model (GAM) fitted line correlating pseudotime values and cancer-specific EMT signature scores. **h** Enrichment UMAP plot of classical EMT markers *Sox4*, *Snai2*, *Col1a1*, and *FN1* in OVCA420 cells treated with TGF-β1. (**i**) Violin plot of NMF program 10 ‘h’ coefficient values for cancer cells in vivo in 16 HGSOC arrayed by immune phenotype. A one-way ANOVA test with Tukey’s multiple comparisons performed on program 10 ‘h’ coefficient values reveals significant differences among all immune subtypes (***p < 0.001).

Our discovery that cancer cells in infiltrated TMEs activate immune regulatory pathways prompted us to assess the EMT status of cancer cells given that cells in partial or fully mesenchymal states are known for their immunoregulatory capabilities^38–40^. Hornburg et al., (2021)^29^ showed that desert TMEs contain malignant cells with higher expression of EMT-associated genes using the MSigDB Hallmark gene set^41^. Our gene set scoring using the subset of 58 Hallmark genes they found associated with the desert tumors similarly reproduced their findings (Supplemental Fig. 3a, b). However, when we performed gene set scoring on the cells with the complete 200-gene Hallmark gene set, no difference was found between tumor immune phenotypes (Supplemental Fig. 3c, d). By applying GSEA enrichment analysis of the three different EMT gene modules, we also found their subset enriched in DEGs from desert derived cancer cells, whereas applying both the full Hallmark EMT gene set and our cancer-specific EMT gene signature yielded negative enrichment scores (Supplemental Fig. 3e; Supplemental Data 1). Based on this analysis, it is unclear if scores calculated using the Hallmark EMT gene set are sufficient to indicate a mesenchymal phenotype. Signature activity among malignant cells from different tumor immune phenotypes revealed some key differences in the cancer cell population (Fig. 3a). The score distribution was then evaluated across the malignant cells derived from the 16 tumors (Fig. 3b) revealing a notably higher EMT activity score of infiltrated tumors compared to excluded and desert tumors (Fig. 3c, d; Supplemental Fig. 4). This result was surprising as desert TMEs have been previously linked to higher EMT signatures in the cancer cells of HGSOC^29,42,43^. Consistent with our finding, individual cancer-specific EMT signature genes such as *PDIA3*, *HLA-A*, *BCAM*, *B2M*, *LGALS3BP*, and *HLA-C* were highly expressed in cancer cells from infiltrated and excluded TMEs (Supplemental Fig. 5). Importantly, none of the tumor samples was found as an obvious outlier for cancer-specific EMT signature scores (Supplemental Fig. 4). Interestingly, cancer cells from infiltrated TMEs appeared to be more heterogeneous in their EMT scoring and skewed towards mesenchymal phenotypes.

To validate the cancer-specific EMT signature’s fidelity in determining EMT, we leveraged an EOC cell line that we have previously shown to undergo transcriptional changes associated with EMT when treated with TGF-β1^44^. OVCA420 cells were treated with TGF-β1 to induce EMT and the cells were collected at three different time-points to perform scRNA-seq (Fig. 3e). We modeled a continuous pseudotemporal EMT trajectory from the data (Fig. 3f) and found that scores from the EMT signature increase throughout EMT progression as expected, alongside pseudotime values (Fig. 3g). Furthermore, the OVCA420 in vitro model of EMT involved activation of classical EMT genes such *SOX4*, *SNAI2*, *COL1A1*, and *FN1* (Fig. 3h). Finally, we leveraged non-negative matrix factorization (NMF), a technique which enables the investigation of coordinated gene expression sources of heterogeneity in the data in a semi-supervised manner with machine learning. We therefore generated a list of 10 possible cell state programs (Supplemental Fig. 6) representing the inherent expression of unique sub-groups of cells throughout the time course (Fig. 3i). Of all the NMF-derived programs, programs 2 and 10 were most consistent with an EMT program. Program 10 (Supplemental Fig. 6) enriches the cell cluster located at the final timepoint of 7-days after TGF-β1 treatment, where we expect a ‘maximal’ mesenchymal state. To further explore program 10, we took the top 500 most-weighted genes in the program and ran an Enrichr analysis (Supplemental Fig. 7a). We discovered that program 10 enriches for Hallmark gene modules related to immunoregulatory pathways such as ‘TNF-alpha signaling via NF-kB’ (***p < 0.001), and others to ‘p53 pathway’ (***p < 0.001), and ‘mTORC1 signaling’ (***p < 0.001) while also enriching the Hallmark EMT gene module. When we performed the same analysis for program 2 (Supplemental Fig. 6), we found reduced enrichment for these same pathways (Supplemental Fig. 7b). These results suggest that a distinct immunoregulatory program is activated upon TGF-β1 induced-EMT and may not be well represented by the Hallmark EMT gene set.

Analysis of the enrichment of cancer cells in the dataset of 16 HGSOC for genes encapsulated in our in vitro-derived NMF program 10 (Supplemental Data 2; Fig. 3i) revealed a greater overall presence of mesenchymal cells in the cancer cell population derived from the infiltrated tumors, followed by cancer cells from excluded and then desert tumors. Scoring the tumors with this defined NMF signature showed enrichment in infiltrated tumors consistent with the distribution of scores from the cancer-specific EMT signature. These findings suggest that program 10 identifies genes related to an immunoregulatory program activated by EMT in HGSOC cancer cells, that is most prevalent in mesenchymal cancer cells from infiltrated tumors. We therefore sought next to determine whether the EMT program could have a direct role in shaping the tumor immune phenotypes.

### EMT is linked to CD8^+^ T cell activity and exhaustion through the LAG3 receptor

**Fig. 4:**
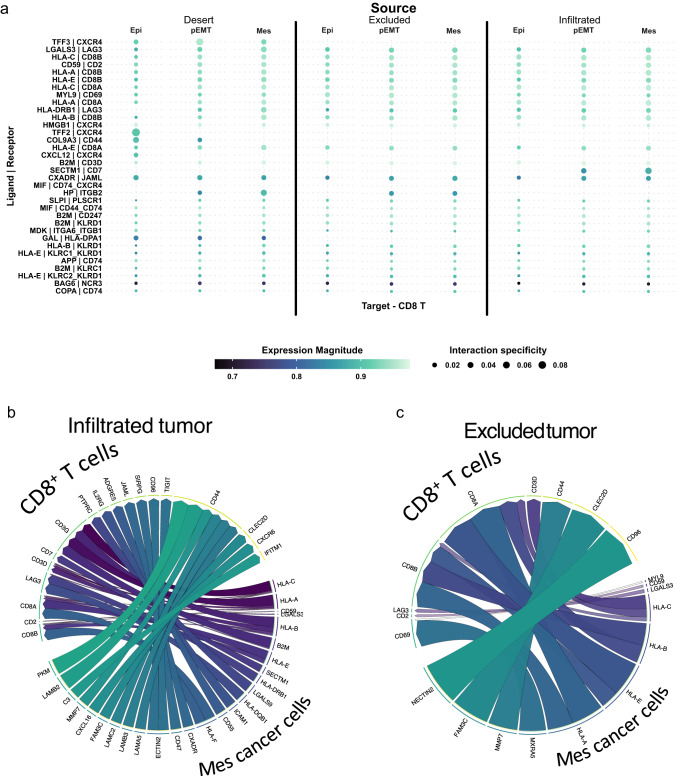
Most ligands targeting CD8^+^ T-cells in the TME originate from the mesenchymal cancer cells. **a** Cell-cell communication analysis of top-most ligand-receptor pairs from cancer cells from 16 HGSOC samples arrayed by infiltration phenotype of tumors and EMT to CD8^+^ T-cells. ‘Expression magnitude’ is a measure of expression levels of a certain interaction. ‘Specificity’ is a measure of the specificity of interactions across all cell types. Interactions are ordered by *p*-value where top-most receptor-ligand pair has lowest *p*-value. Circle plot of ligands originating from mesenchymal cancer cells in infiltrated tumors (**b**) and excluded tumors (**c**), and the receptors targeted on recipient CD8^+^ T-cells. Order and color of ligands corresponds to *p*-value aggregate rank reflecting specificity of interactions (most specific – purple color, clockwise). The number of tracks per ligand corresponds to the number of overall interactions the ligand has with different receptors. Track width corresponds to *p*-value aggregate rank within a ligand if the ligand has multiple receptor targets.

Since our findings indicate that EMT in cancer cells correlates with the presence of immune cells (Fig. 3d; Supplemental Fig. 5a), we dissected the inter-cellular communications in each tumor immune phenotype by evaluating the unique expression of ligands in malignant cells and their cognate receptors in CD8^+^ T cells. CD8^+^ T cells are very abundant in infiltrated tumors (Fig. 1e, Supplemental Fig. 1), and we were interested in assessing whether EMT influences antitumor immunity in infiltrated and excluded cancers. The ligand-receptor interactome analysis of communication between cancer cells from infiltrated and excluded tumors to CD8^+^ T-cell receptors revealed that the majority of interactions are borne from mesenchymal cancer cells towards CD8^+^ T cells in both immune phenotypes (Supplemental Fig. 8a, b). We then assessed the top cancer cell to CD8^+^ T cell receptor-ligand interactions with the former arrayed by EMT status (Fig. 4a). Heterogeneity in signaling between different immune phenotypes is evident, where receptor-ligand pairs such as *SECTM1 - CD7* appear to be unique to infiltrated tumors (Fig. 4a). The top five ligands targeting CD8^+^ T-cells by mesenchymal cancer cells from infiltrated tumors were *HLA-C*, *HLA-A*, *CD59*, *LGALS3*, and *B2M* (Fig. 4a, b). We found similar gene patterns in mesenchymal cells’ ligands in excluded tumors with *CD59*, *LGALS3*, *HLA-C*, *HLA-B*, and *HLA-E* (Fig. 4a, c). The finding of classic MHC I molecule expression by cancer cells was not surprising as HLA I expressing tumors have been previously linked to TIL frequency, with TILs eventually inducing HLA allotype selection, consequently enabling cancer cells to evade antitumoral immunity^45,46^. Nonetheless, we were expecting to find other dominant inhibitory ligands such as PD-L1 promoting immunosuppression, but *LGALS3* (Galectin-3, Gal-3) emerged as a top ligand, highlighting its potential role in the EMT driven immunosuppression in EOC^9,47–50^.

Further investigation of the specific receptors targeted by *LGALS3* showed that the *LAG3* complex on CD8^+^ T cells is the primary recipient of *LGALS3*, as well as MHC II molecules, matching previous literature findings^51–53^ (Fig. 4a−c; Supplemental Fig. 8c, d). In fact, *LAG3* appears to be one of the most targeted receptor complexes by rank in both infiltrated and excluded tumors, suggesting the possible exhaustion of these cells. The observation linking the LGALS3-LAG3 interaction to EMT prompted us to further investigate CD8^+^ T cell exhaustion as a possible consequence of the EMT process in primary HGSOC.

**Fig. 5:**
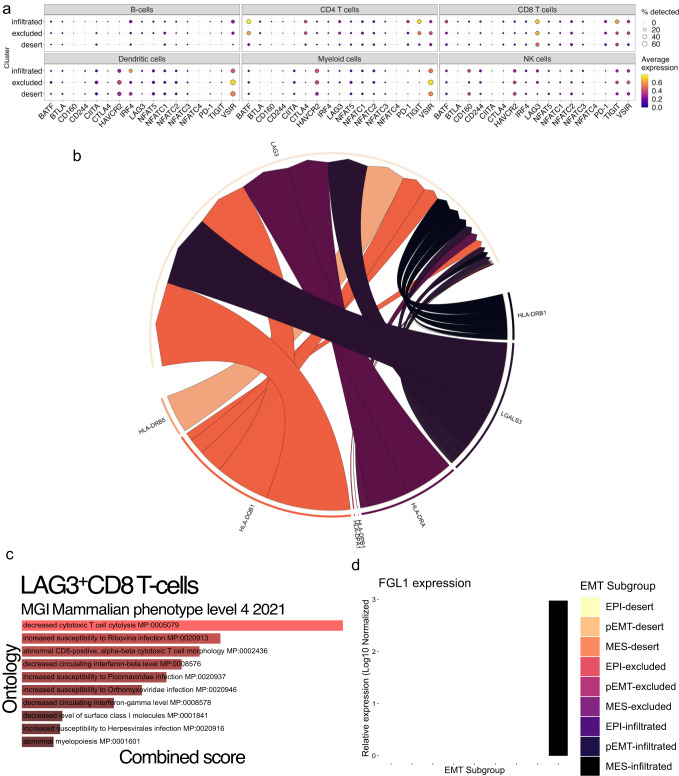
Most CD8^+^ T-cells in HGSOC express *LAG3*, a marker of T cell exhaustion. **a** Dot plot showing expression levels and frequency of common markers of T-cell exhaustion in the entire CD45^+^ cell compartment. **b** Circle plot of CD8^+^ T-cell LAG3 receptor complex and all the ligands that target it. **c** Analysis of enriched pathways from a DEG analysis of LAG3^+^ CD8 T-cells compared to LAG3^-^ T-cells in the Mouse Genome Informatics (MGI) database. **d** Log-normalized expression of FGL1 in the EMT-delineated compartment of cancer cells from each tumor immune phenotype.

Common exhaustion markers or checkpoint inhibitors of CD8^+^ T-cells include *BTLA*, *CD160*, *CD244*, *CTLA4*, *HAVCR2*, *LAG3*, *TIGIT, and PD1*, the *NFAT* family *(NFAT5*, *NFATC1-4)* of transcription factors involved in promoting T cell exhaustion, and *IRF4, BATF, VSIR, and CIITA*, a master regulator of MHC II expression which is known to interact with LAG3. Of these markers, *LAG3* and *TIGIT* are most active in CD8^+^ T cells in infiltrated tumors (Fig. 5a). In fact, high *LAG3* and *TIGIT* expression in infiltrated tumors could be indicative of a specific inflammatory milieu triggering *LAG3* upon CD8^+^ T cell infiltration and sensitizing these cells to exhaustion signals from mesenchymal cancer cells. A breakdown of all the ligands targeting the *LAG3* receptor on CD8^+^ T cells, regardless of cancer cell EMT status or tumor immune phenotype, showed that most ligands for *LAG3* are HLA II molecules, with *LGALS3* being the only non-MHC related ligand (Fig. 5b). Despite HLA II ligands making up the bulk of interactions between cancer cells and *LAG3* on CD8^+^ T-cells, we did not find any associations between EMT and HLA II expression (data not shown). Additionally, NECTINs 2, 3, and 4 are the major interacting partner with TIGIT between cancer cells and CD8^+^ T-cells, suggesting another possible vector for T cell exhaustion by mesenchymal cancer cells.

GSEA of the DEGs between LAG3^+^ and LAG3^-^ CD8 T cells revealed enrichment for molecular pathways related to decreased cytotoxic T cell cytolysis (MP:0005079) and abnormal CD8^+^ T cell morphology (MP:0002436) (Fig. 5c), suggesting that there is a significant contingent of CD8^+^ T cells in infiltrated tumors that are cytolytically non-functional as a result of LAG3-related signaling. Fibrinogen-like protein 1 (FGL1) has been shown recently to be a ligand of LAG3, also potentially influencing T cell exhaustion^54^. Interestingly, we found FGL1 is expressed only by mesenchymal cancer cells in infiltrated tumors (Fig. 5d). As our findings show that there is a significant proportion of LAG3^+^ CD8^+^ T-cell targeted by ligand LGALS3^+^ originating from mesenchymal cancer cells, we sought to further explore the link between *LGALS3* and EMT to better elucidate how EMT could modulate CD8^+^ T-cell exhaustion.

### LGALS3 is linked with EMT in both in vivo and in vitro contexts

**Fig. 6:**
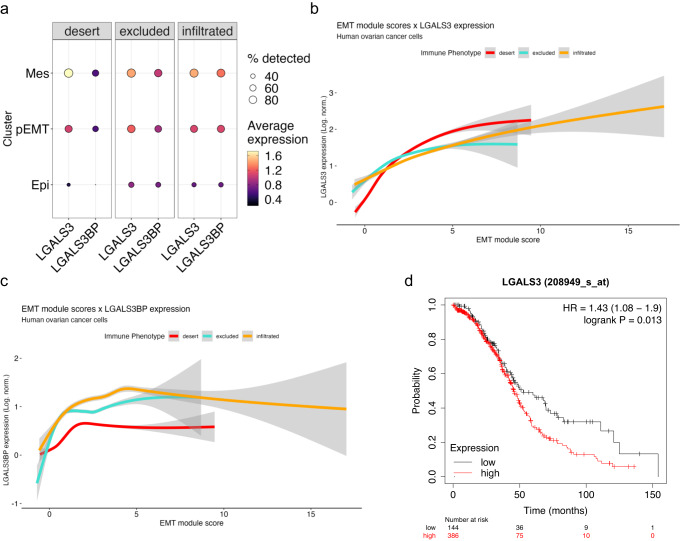
*LGALS3* and *LGALS3BP* expression in cancer cells correlates with EMT in vivo. **a** Dot plot showing *LGALS3* and *LGALS3BP* in the cancer cell compartment arrayed by EMT status and the percentage of cells that express these genes in each immune phenotype. Log-normalized *LGALS3* (**b**) and *LGALS3BP* (**c**) expression fitted via generalized linear model against cancer-specific EMT signature scores in cancer cells, segregated by tumor immune phenotype. **d** Kaplan−Meier curve comparing patient overall survival in *LGALS3*-high and *LGALS3*-low expressing ovarian tumors from TCGA data.

To determine whether *LGALS3* has any association with EMT, we examined its expression across cancer cells arrayed by both tumor immune phenotype and EMT status. We also assessed LGALS3 Binding Protein (*LGALS3BP*) as it is known to synergize with *LGALS3* in certain contexts^55^. Both *LGALS3* and *LGALS3BP* appear to have higher expression in mesenchymal cancer cells, regardless of the tumor immune phenotype (Fig. 6a). We fitted expression of *LGALS3* (Fig. 6b) and *LGALS3BP* (Fig. 6c) against EMT score and arrayed by tumor immune phenotype to find that *LGALS3* expression increases alongside the cancer specific EMT signature expression in every tumor immune phenotype and every delineated EMT phase (i.e., EPI, pEMT, and MES). By contrast, *LGALS3BP* increases most in earlier, epithelial phases in all tumor immune phenotypes, and then plateaus, suggesting it is more important in earlier phases of the EM program. To determine whether *LGALS3* correlates with survival in HGSOC, we analyzed the TCGA data and found that low *LGALS3* expression in bulk RNA-Seq of HGSOC is associated with longer survival (*p = 0.013) (Fig. 6d). These findings suggest that *LGALS3* expression correlates with EMT in cancer cells and contributes to reduced survival of ovarian cancer patients.

**Fig. 7:**
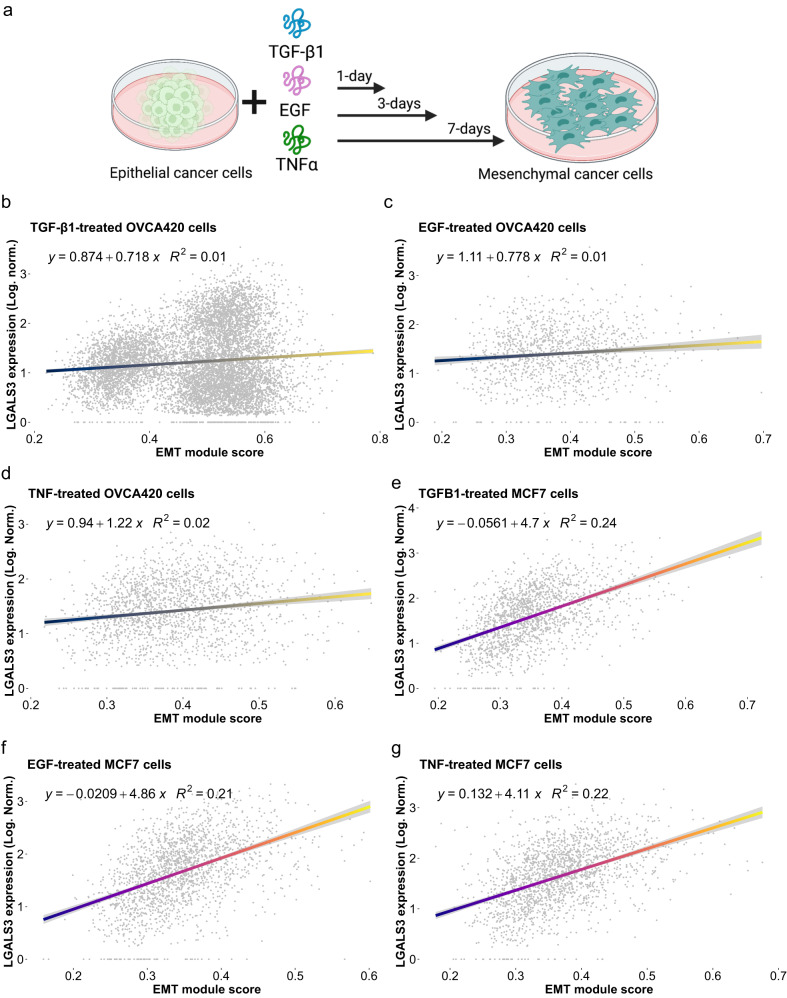
LGALS3 is directly correlated with the EMT in vitro in ovarian and breast cancer cell lines. **a** Schematic of cell line treatment (**b**−**d**) Log-normalized expression of LGALS3 as generalized linear model against cancer-specific EMT signature scores in OVCA420 ovarian cancer cells treated with TGF-β1 (**b**), TNFα (**c**), and EGF (**d**). Log-normalized expression of LGALS3 GLM-correlated against cancer-specific EMT signature scores in breast cancer MCF7 cells treated with TGF-β1 (**e**), TNFα (**f**), and EGF (**g**).

To elucidate if *LGALS3* is associated with the EMT in other model systems and under different conditions, we referred to our TGF-β1 treated OVCA420 cancer cells and correlated expression of *LGALS3* with the cancer specific EMT signature scores (Fig. 7a). We fitted a generalized linear model and noted a modest positive association (R^2^ = 0.01) between *LGALS3* and cancer specific EMT signature scores (Fig. 7b). We noted that it appeared as though there are two populations of mesenchymal cells where *LGALS3* is expressed in one but not the other. To determine if *LGALS3* is consistently implicated in EMT rather than simply a consequence of TGF-β1 treatment, we accessed our previously published data^44^ where multiple cell lines of different cancer types (ovarian, OVCA420; breast, MCF7; prostate, DU145; and lung, A549) were each treated with EMT inducers (TGF-β1, TNFα, EGF). While these proteins were chosen for their ability to induce EMT through different receptors and pathways, in certain cancers they can be secreted by immune cells, such as TNFα by CD8^+^ T cells^56^, thus mimicking the immune response to cancer cells. We found a similar positive correlation between *LGALS3* and cancer-specific EMT signature scores in OVCA420 cells treated with either EGF (R^2^ = 0.01) or TNF-α (R^2^ = 0.02) (Fig. 7c, d). Other cancer cell types also showed positive correlations: MCF7 cells (TGF-β1, R^2^ = 0.24; TNF-α, R^2^ = 0.21, EGF, R^2^ = 0.22) (Fig. 7e−g), DU145 (TGF-β1, R^2^ = 0.1; TNF-α, R^2^ = 0.11, EGF, R^2^ = 0.11) (Supplemental Fig. 9a−c), and A549 (TGF-β1, R^2^ = 0.05; TNF-α, R^2^ = 0.1, EGF, R^2^ = 0.12) (Supplemental Fig. 9d−f), with breast cancer cells showing the strongest correlations between *LGALS3* expression and EMT. Taken together, our findings indicate that *LGALS3* expression is correlated with the EMT of cancer cells in vivo and in vitro, and that EMT drives expression of *LGALS3* in HGSOC cells. Additionally, *LGALS3* expression is a “core” gene in the EMT program that is activated under a variety of EMT inducers through TGF-β1-, or TNF-α-, or EGF-induced signaling. Mesenchymal cancer cells in turn exert inhibitory antitumoral signaling on CD8^+^ T cell promoting a dysfunctional cytotoxic state and exhaustion by binding to the *LAG3* receptor complex.

### GSK3 and Aurora-A kinase inhibitors attenuate LGALS3 expression

**Fig. 8:**
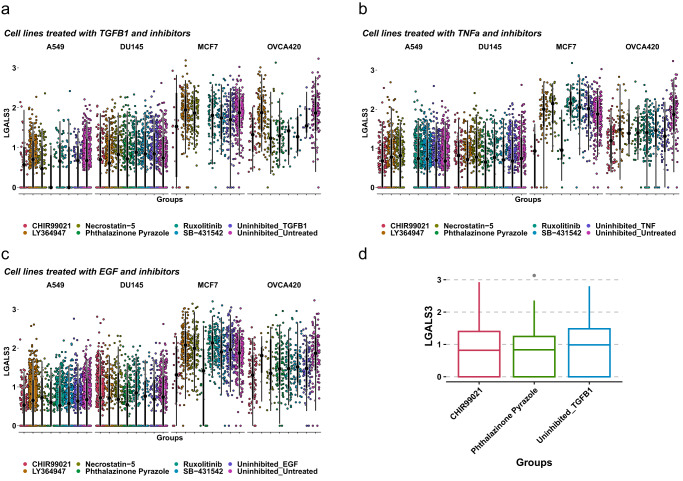
Kinase inhibitor screen shows GSK3 and Aurora-A kinase inhibitors attenuate EMT and LGALS3 expression. Log-normalized expression of LGALS3 in A549, DU145, MCF7, or OVCA420 cell lines either treated either with EMT inducers TGF-β1 (**a**), TNFα (**b**), EGF (**c**) or EMT inducers and kinase inhibitors: RIP1 kinase inhibitor Necrostatin5, TGFβR1 kinase inhibitor LY364947, JAK1/2 kinase inhibitor Ruxolitinib, TGFβ/ALK kinase inhibitor SB-431542, GSK3 kinase inhibitor CHIR99021, or TGF-β1 and Aurora kinase A inhibitor phthalazinone pyrazole. **d** LGALS3 expression in OVCA420 cells treated either with TGF-β1, TGF-β1 and GSK3 kinase inhibitor CHIR99021, or TGF-β1 and Aurora kinase A inhibitor phthalazinone pyrazole.

To determine the possible pathways linking EMT and *LGALS3* expression we referred to a previously published kinase inhibitor screen that elucidated some of the signaling dependencies of the EMT^44^. Particularly, the modularity of the EMT spectrum was revealed to be a core part of the process where some TGF-β1R-independent kinases attenuated the EMT despite treatment with various inducers (TGF-β1, TNFα, and EGF). In the original screen it was discovered that both RIP1 kinase inhibitor Necrostatin-5 and TGF-β1R inhibitor LY364947 abrogated EMT in all treatment conditions. When we explored this screen further (Fig. 8a−c), we noted that two kinase inhibitors produced the most consistent reduction in *LGALS3* expression across cell lines and treatments: CHIR99021, a GSK3 kinase inhibitor and phthalazinone pyrazole, an Aurora kinase A inhibitor (Fig. 8d), suggesting *LGALS3* expression is potentially dependent on activation of the Wnt and NF-kB pathways, or the β-catenin pathway during EMT. Finally, when we explored the effects of these two kinase inhibitors on pseudotime values as a measure of EMT and LGALS3 expression, we found that neither CHIR99021 nor phthalazinone pyrazole appear to attenuate the EMT, suggesting that blocking LGALS3 upregulation is not sufficient to prevent EMT (Supplemental Fig. 10).

## Discussion

In this study, we show that the EMT, a complex cellular process underpinning metastasis, associates with *LGALS3* expression, which may act as ligands for *LAG3* in CD8^+^ T cells, promoting T cell exhaustion and dampening antitumor immunity. Additionally, we show that infiltrated TMEs in HGSOC have cancer cells that are, on average, more mesenchymal than those found in the excluded or desert phenotypes, with the potential implication that a greater chance for metastasis and immunosuppression is possible in those tumors. Given that infiltrated and excluded tumors have been correlated with better survival among HGSOC patients compared to desert tumors, and the presence of CD8^+^ T cells in these tumors, there is great potential for treating these patients with LAG3 checkpoint blockade therapy combined with other immune checkpoint inhibitors as it has been demonstrated in mouse IE9mp1 EOC model by Huang et al. (2015)^22^. Recently, the US Food and Drug administration (FDA) approved second-generation checkpoint inhibitor ‘Opdualag’, an anti-LAG3 and anti-PD1 combination drug that targets metastatic or unresectable melanoma^57^. Additionally, the development of new peptides, such as C25, that block LAG3 binding to MHC II has been proven to activate CD8^+^ T cells^52,58^. LAG3 blockade therapies have been shown to have therapeutic benefit for patients with chronic lymphocytic leukemia, melanoma, and pancreatic adenocarcinoma^59,60^. In *BRCA-*mutated HGSOC patients, LAG3 was positively correlated with PD-L1; however, combination immunotherapies in human HGSOC to block the activity of both inhibitory checkpoints were found to have negligible efficacy, suggesting other underlying mechanisms governing the immunosuppressive TME of HGSOC in the context of *LAG3* expression^47^.

Recent scRNA-Seq investigations on BRCA1/2 mutated HGSOCs have showed a potential link between EMT and CD8^+^ T-cell exhaustion^61^. As well, EMT and T cells found in malignant ascites from EOC express high levels of LAG3 and PD-1^62,63^. This latter observation is of particular interest as it has been previously suggested that EOC ascites contain a significant mesenchymal cancer cell population^64,65^. As well, mesenchymal and pEMT cancer cell states have been linked to higher PD-L1 expression in breast cancer^66^ suggesting that even a pEMT state is correlated with immunosuppression. Similar relationships between LGALS3 and T cell exhaustion in TGF-β1 induced fibrotic disease^67^ have been uncovered, where LGALS3 inhibitors^68,69^ have proven to be effective in treating the disease. In cancer contexts where TGF-β1 plays a major role in initiation and maintenance of EMT, patients may also benefit from LGALS3 inhibitors. For example, LGALS3 has a strong proinflammatory role when expressed by fibroblasts^70^, eliciting secretion of IL-6, CXCL8, CCL2, and CCL5 all of which are factors that play a role in carcinogenesis and immunosuppression. Additionally, anti-TIGIT therapy has shown mixed success in treating a variety of solid tumors, including ovarian cancer^71^ with other clinical trials demonstrating better clinical benefits in tumors with high *TIGIT* expression^72^. Curiously, CD8^+^ T cells infiltrated ovarian tumors have high *TIGIT* expression as shown in this paper (Fig. 5), which may suggest ovarian tumors are a potential target for combination anti-LAG3 and anti-TIGIT therapy. Moreover, combination therapies including anti-TIGIT and anti-PD-L1 synergize to enhance cytolytic CD8^+^ T cell activity^73,74^ which could be used to target ovarian tumors of the infiltrated subtype to alleviate exhaustion and immunosuppression.

Galectin-3 (Gal-3/LGALS3), encoded by the *LGALS3* gene, is a lectin that can be both expressed on the cell surface^75^ and secreted^76^, and is expressed by the majority of human cells. It exhibits several immune-regulatory functions such as reducing the affinity of TCR for its cognate MHC I-peptide ligand by sequestering the TCR from its CD8^+^ co-receptor^77^, causing apoptosis^78^, and internalization of the TCR^79^, leading to decreased interferon-gamma (IFN-γ) production upon LAG3 engagement on CD8^+^ T cells^51^. *LGALS3* has been linked to poor prognosis in EOC^80^ with the assertion that it may be activating the Wnt/β-Catenin pathway to effect cancer stemness mechanisms^81^. Additionally, overexpression of Galectin-1 (*LGALS1*), a protein similar to *LGALS3*, promotes EMT in fibroblasts through TGF-β signaling pathways^82^. *LGALS3* has also been linked to the EMT previously^83^ and has been suggested as a T cell-directed immunotherapy to increase efficacy of current immune checkpoint inhibitors. Interestingly, some of our in vitro results demonstrate there could be two different EMT trajectories where *LGALS3* is expressed in one but not the other, suggesting that the EMT can lead to heterogenous populations of mesenchymal cells.

The potential to rescue CD8 + T-cells from exhaustion has been shown, notably in studies where anti-PD1 therapy was shown to improve the function of exhausted tumor infiltrating CD8^+^ T cells in ovarian cancer^84^. Whether targeting LGALS3-LAG3 axis has similar potential to rescue CD8 + T cell function alone or in combination with other immunotherapies such as anti-PD1, anti-CTLA4, and anti-TIGIT, requires further investigation. The *LGALS3* inhibitor GB0139 has shown promise in acute lung injury where its mechanism of action involves reducing IL-6, TNF-α, and MIP-1α^69^, and thus may also prove efficacious in ovarian cancer where TNF-α plays a role in EMT initiation and maintenance. Galectin-3C, a dominant-negative inhibitor of *LGALS3*, reduces the metastatic potential of ovarian cancer either in combination with Paclitaxel or alone^85^.

It should be noted that the suggestion to target infiltrated ovarian tumors for checkpoint therapy is based on our interpretation of data from primary tumors. Indeed, the immune environment in the ascites or metastases may be different where CD8 + T cells may not be exhausted and could clear cancer cells, thus reducing metastatic spread and contributing to the positive survival prognosis of infiltrated tumors. In these cases, checkpoint therapy may be less effective depending on the TME but could still assist the immune system in clearing the primary tumor site.

In our screen of various kinase inhibitors, we found the highly selective and potent GSK3 inhibitor CHIR99021 reduced expression levels of *LGALS3* while not affecting EMT signature scores, suggesting it may be suitable for therapeutic investigation due to its possible specificity to *LGALS3*. GSK3 inhibition or downregulation can potentiate the cytotoxicity of CD8 + T cells against lymphoma cells^86^, gastric cancer cells^87^, and melanoma cells, with the latter also showing a blockage of LAG3 because of GSK3 targeting by small molecule inhibitors^88^.

**Fig. 9:**
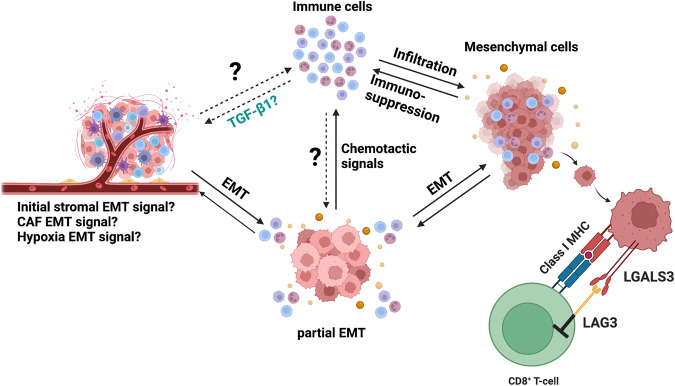
Schematic of hypothesized positive feedback loop between immune cells and cancer cells leading to metastasis and immunosuppression. In cancer, mesenchymal cancer cells have been shown to be associated with immunosuppression and exhaustion of T-cells in the TME. In our paper, we demonstrate one way in which mesenchymal cells may exhaust CD8^+^ T-cells in the TME of epithelial ovarian cancer. Additionally, we show there is an association between infiltration of immune cells, particularly T-cells, and EMT. It is currently unknown whether cancer cells instigate the infiltration of T-cells after undergoing EMT, or whether the initial infiltration of T-cells and other immune cells triggers EMT in cancer cells. We propose this model as a potential future direction, where the relationship between immune cell infiltration and EMT can be further investigated.

A surprising finding in this study is the correlation between EMT and tumor immune cell infiltration, since previous studies have reported greater EMT in desert tumors. Certain immune cells, such as CD4^+^ CD25^+^ T_reg_ cells, tumor-associated macrophages, and myeloid-derived suppressor cells (MDSCs) can induce EMT in cancer cells^8^ and our study shows upregulation of chemotactic signals during EMT. Consequently, we propose the existence of a positive feedback loop between immune cells and cancer cells, based on their EMT status. In this scenario, initial signals favoring EMT are propagated through the TME, perhaps from the stroma and fibroblasts^89–91^, or the result of hypoxic conditions^92–94^. Cancer cells receiving those signals undergo EMT and upregulate pathways related to leucocyte and lymphoid chemotaxis by secreting relevant interleukins and chemokines, as predicted by our findings. Tumor infiltration by activated immune cells promote secretion of factors such as TGF-β1, TNF-α, and ADAM17 intensifying EMT signals and further driving EMT progression in the cancer cell population, resulting in tumor-promoting positive feedback loop. This would ultimately lead to T cell exhaustion through elevated LAG3 stimulation, among other coinhibitory markers, such as TIGIT (Fig. 9). The validity of this feedback loop hypothesis warrants further investigation, for example, by assessing the tumor immune cell composition in mouse models of ovarian cancer that express an inducible EMT signal, such as *SNAIL*.

Our results do not eliminate the possibility that active CD8 + T cells select for survival of pro-exhaustion mesenchymal cells by successfully attacking the less immunosuppressive epithelial cells. However, this still leaves a question about the origin of signals that promote EMT, whether they originate from another population of cells such as fibroblasts secreting TGF-β1. Our results demonstrate that while desert tumors have a large fibroblast population, there are fewer mesenchymal cancer cells in them than infiltrated tumors, further suggesting that EMT signals likely originate from a different cell population, such as immune cells. Therefore, even if CD8 + T cells and other immune cells select for mesenchymal cells, the trigger for EMT may also originate in those cells.

Our results indicating that there are more mesenchymal cells in infiltrated TMEs as opposed to desert TMEs likely differ from Hornburg et al. ^29^ for a few reasons. First, we used a cancer-specific EMT signature that can capture the intra-tumoral heterogeneity of the epithelial/mesenchymal program and states. The Hallmark EMT gene set, when combined with classical EMT markers (i.e., *SNAI1/2, ZEB1/2, TWIST1*) captures mostly pEMT states^95^ and is based on founder gene sets, some of which are not from a cancer context^41,96^. Second, the Hallmark EMT gene set enriches in stromal compartments^97^ and also in cancer-associated fibroblasts^95^ suggesting it may be poorly optimized to capture malignant mesenchymal cells at all, at least on a single-cell RNA level. Only 58 out of 200 genes in the Hallmark EMT gene set were enriched in Hornburg et al.’s analysis of desert tumors and were on the cusp of significance. Third, our scoring strategy to compute EMT scores differs from their enrichment analysis in that our strategy provides valuable single-cell level scoring compared to bulk sample averages. Pseudo-bulking scoring methods have difficulty accounting for cell-to-cell variability^98^ which could impact EMT calculations where different cell states exist even within the same cell type. We believe that as the cancer-specific EMT signature was derived from scRNA-Seq datasets, it may be best used in single-cell contexts using analysis algorithms best-suited to the technology.

With single-cell sequencing technologies still rapidly improving in both capability and fidelity, there is tremendous potential to expand the study of tumor interactomes either through advances in technology or improved algorithm design. For example, technologies such as CITE-Seq can be leveraged to supplement genomic sequencing data with cell surface-level protein expression data combined with established receptor-ligand algorithms to generate a clearer picture of the interactome of the TME^99^. Future exploration of the interactome that we have generated could reveal novel interactions between cancer cells and other immune cell types, or further delve into subtypes of T cells such as T_reg_ cells, to paint a more complete picture of the receptor-ligand interactions in the TME of HGSOC. To conclude, ovarian mesenchymal cancer cells suppress CD8^+^ T cell activity through the pro-exhaustion LAG3-LGALS3 pathway. There is a therapeutic opportunity to target HGSOC that are already infiltrated by CD8^+^ T cells and relieve them of barriers that dampen antitumoral activity, such as T cell exhaustion. Other than targeting the LAG3-LGALS3 pathway itself, it may be possible to attenuate the EMT to prevent pathway activity specifically and remove a possible source of immunosuppression.

## Methods

### Cell culture

The human ovarian cancer cell line OVCA420 was kindly provided by Dr. Gordon Mills. Cells were cultured in Dulbecco’s Modified Eagle Medium (DMEM) with 4.5 g/L glucose, L-glutamine, and sodium pyruvate (Corning, 10-013-CV), supplemented with 10% of fetal bovine serum (FBS) and cultured at 37 ^o^C with 5% CO_2_.

### OVCA420 scRNA-seq EMT time course experiment

OVCA420 cells (10,000/well) were plated into in 6-well plates. Cells were treated with 10 ng/mL TGF-β1 (R&D Systems, #240-B-010), with treatment timed in such a way that all time-points were synchronized at the time of collection. Cells were passaged as needed to avoid confluence, and fresh TGF-β1 was added every two days with refreshed media. Cells were not passaged in the 2 days prior to final collection to avoid artifacts during sequencing. Single-cell suspensions were processed using the 10x Genomics Single Cell 3’ RNA-seq kit v3. Final libraries were sequenced on an Illumina HiSeq 4000 after gene expression libraries were prepared according to the manufacturer’s protocol. Raw sequencing reads were processed using CellRanger v2.0.1 using the GRCh38 build of the human genome and default parameters. Graphics were created using ‘ggplot2’ and ‘SCpubr’^100^.

### Data quality control and processing

Quality control was first performed independently on each 10x Genomic library, and all main processing steps were performed with Seurat V3^101^ for the OVCA420 cells and Seurat v4^102^ for the ovarian cancer datasets. Expression matrices for the OVCA420 cells were imported into R as Seurat objects. Only cells with more than 200 genes detected were retained and cells with a high percentage of mitochondrial gene expression were also removed. For the OVCA420 treatment time course, an independent Seurat object was made combining all timepoints, followed by a standard workflow by first removing genes detected in fewer than 1% of the cells for each timepoint. We then obtained the top 2000 most variable genes using the ‘vst’ selection method in Seurat, scaled RNA expression values and regressed out mitochondrial reads, total UMI count, and cell cycle scoring. Cell cycle regression was handled by ‘SCTransform’^103,104^, which was also used to normalize the RNA matrices for each sample using regularized negative binomial regression. After this, PCA was run on the variable genes and all UMAP embeddings were calculated from the first 30 principal components.

For the ovarian cancer datasets^42^, each individual tumor sample matrix was obtained as tables divided into stroma, CD45^+^, and tumor cell files that we first made into Seurat objects with a minimum of 200 genes per cell and then merged using the ‘base::merge()’ function prior to processing, yielding 16 individual samples (i.e., 16 tumors). Each sample was processed independently similarly to the OVCA420 cells. Briefly, cells with high percentage mitochondrial genes and low feature number were subset out and cell cycle genes were regressed out for each sample using ‘SCTransform’^103,104^. ‘SCTransform’ was also used to normalize the RNA matrices for each sample using regularized negative binomial regression. PCA was then applied to each individual sample and UMAP embeddings were calculated from the first 30 principal components. We also added metadata such as immune phenotype and patient-ID in each samples’ Seurat metadata slot.

### Semi-supervised cell labeling and integration

Cell type labeling was first performed using common markers for cancer cells (*KRT19*, *AMHR2*, *ELF3*, *EPCAM*) and fibroblasts (*COL1A1*, *COL1A2*). We also labeled endothelial cells (*CDH5*, *CLDN5*) and smooth muscle cells (*ACTA2*) to find very small populations (<100 cells) for each, so we removed these populations. To label the CD45^+^ population we applied Seurat’s multimodal reference mapping method^102^ to each individual sample with their published CITE-Seq reference object of 162,000 PBMCs measured with 228 antibodies. We used all default settings provided by Seurat for this part of the processing pipeline. After mapping the cell populations by the reference object, we merged this with our labels for cancer cells and fibroblasts to finalize the individual sample objects with labels for all clusters.

Seurat’s integration^101^ was used to align and combine shared populations across the 16 tumors. Briefly, Seurat matches pairs of cells across datasets that share certain biological states, or anchors, based on bulk RNA expression. The corrected data was then scaled and UMAP embeddings were applied to it based on 30 principal components from a PCA run.

### EMT scoring strategy

For EMT scoring, we subset the cancer cell population from the integrated object and used the ‘AddModuleScore()’ Seurat function together with our previously published cancer-specific EMT signature^96^ to assign an EMT score to each cancer cell. This EMT signature reflects the most consistent expression patterns associated with epithelial-mesenchymal plasticity in cancer^96^. EMT scoring was similarly performed for the OVCA420 object. We then calculated the mean of each individual samples’ EMT scores and labeled every cell that fell under a threshold of ‘mean–1 standard deviation’ an epithelial cell and every cell above a threshold of ‘mean+1 standard deviation’ a mesenchymal cell, with all cells in-between labeled as partial-EMT (pEMT).

### Differential gene expression analysis

The Wilcoxon rank sum test implemented in the ‘FindMarkers()’ or ‘FindAllMarkers()’ functions of Seurat were used to calculate all differentially expressed genes between the input populations. For volcano plots and analysis of most differentially expressed genes we used a cutoff of p-adjusted < 0.05 and for log2 fold-change (log2fc) the mean of the log2fc ± 2*standard deviation of log2fc.

### Gene set enrichment analysis

Gene set enrichment analysis (GSEA) was performed using the ‘fgsea’ R package^105^. Input genes were ranked by their log2 fold-change values. Reference gene sets were collected from the Molecular Signatures Database (MSigDB) v6.2. For gene set enrichment of LAG3^+^-high CD8^+^ T-cells, we used the Enrichr^106–108^ online tool (https://maayanlab.cloud/Enrichr/).

### Pseudotemporal ordering of cells

For a detailed explanation of the pseudotime pipeline, refer to Cook and Vanderhyden (2020)^44^. Briefly, we used R package ‘psupertime’^109^ v0.2.6 (https://github.com/wmacnair/psupertime) to calculate pseudotime scores on the top 3000 most variable genes for the OVCA420 Seurat object. Psupertime requires scRNA-seq data with ordinal labels to build a linear combination of genes that vary consistently over the time course and are used to assign a pseudotemporal value to individual cells. Individual cells with pseudotemporal values were correlated with other genes or modules (eg. EMT signature scores).

### Non-negative matrix factorization

Non-negative matrix factorization (NMF) was performed using the ‘RcppML’ R package^110^. Briefly, ‘RcppML’ leverages NMF as a machine learning strategy to learn coordinated gene activity in sparse data and present summaries of biological processes as broken into individual vectors of weighted values that contribute to the overall dimensionality in the data.

### Cell-cell communication analysis

For cell-cell communication analysis, we used LIANA^111^, a tool that integrates multiple methods for cell-cell communication inference in single-cell data. LIANA provides a consensus-based rank aggregate for receptor-ligand pairs from the results of multiple cell-cell communication algorithms through ‘robust rank aggregation’ (RRA). Briefly, we chose the default settings of LIANA for our analysis that use methods from SCA, NATMI, Connectome, CellPhoneDB, and CytoTalk to evaluate receptor-ligand pairs. We ran LIANA using the function ‘liana_wrap()’ on our integrated object. The cancer cell population was divided into unique identities based on immune phenotype and EMT score, resulting in 9 identities: Infiltrated-epithelial, Infiltrated-pEMT, infiltrated-mesenchymal, excluded-epithelial, excluded-pEMT, excluded-mesenchymal, desert-epithelial, desert-pEMT, desert-mesenchymal. We then aggregated all the methods into a single matrix using ‘liana_aggregate()’ to construct maps of cell-cell communications with ‘Circlize’^112^ based on the top ‘aggregate_rank’ of receptor-ligand pairs.

### Kaplan-Meier plots

For KM plots, https://kmplot.com/^113^ was accessed to use bulk RNA-Seq TCGA and microarray data to construct KM survival plots. For *LAG3* and *LGALS3*, we plotted overall survival of optimally debulked patients with high-grade (3 + 4), later stage (2 + 3 + 4) tumors with *TP53* mutation. We chose these settings because they match well with the patient data from Hornburg et al. (2021)^29^ and represent a subset of some of the worst HGSOC samples based on expected patient outcomes.

### Reporting summary

Further information on research design is available in the Nature Research Reporting Summary linked to this article.

### Supplementary information

Reporting summary

Supplemental Figures

Supplemental Data 1

Supplemental Data 2

## Data Availability

Sixteen high-grade serous ovarian cancer datasets were obtained with permission from European Genome-Phenom Archive (EGAD00001006974). For kinase inhibitor-treated time-course experiment, raw sequencing files and processed UMI count matrices have been obtained from the NCBI Gene Expression Omnibus under the accession GSE147405. For OVCA420 time course treated with TGF-β1 experiment, raw sequencing files and processed UMI count matrices have been deposited in the NCBI Gene Expression Omnibus under the accession GSE247098.
