# Supplementary material for: Mesenchymal ovarian cancer cells promote CD8+ T cell exhaustion through the LGALS3-LAG3 axis
Source: NPJ Syst Biol Appl. 2023 Dec 12;9:61. doi: 10.1038/s41540-023-00322-4 (PMC10716312; doi:10.1038/s41540-023-00322-4)
Supplement: Supplementary file 1 — Reporting summary [file 41540_2023_322_MOESM1_ESM.pdf]

nature portfolio

Corresponding author(s): Edward Yakubovich

Last updated by author(s): Nov 6, 2023

# Reporting Summary

Nature Portfolio wishes to improve the reproducibility of the work that we publish. This form provides structure for consistency and transparency in reporting. For further information on Nature Portfolio policies, see our Editorial Policies and the Editorial Policy Checklist.

## Statistics

For all statistical analyses, confirm that the following items are present in the figure legend, table legend, main text, or Methods section.

| n/a | Confirmed | |
|---|---|---|
| ☒ | ☐ | The exact sample size (*n*) for each experimental group/condition, given as a discrete number and unit of measurement |
| ☒ | ☐ | A statement on whether measurements were taken from distinct samples or whether the same sample was measured repeatedly |
| ☐ | ☒ | The statistical test(s) used AND whether they are one- or two-sided<br>*Only common tests should be described solely by name; describe more complex techniques in the Methods section.* |
| ☒ | ☐ | A description of all covariates tested |
| ☐ | ☒ | A description of any assumptions or corrections, such as tests of normality and adjustment for multiple comparisons |
| ☐ | ☒ | A full description of the statistical parameters including central tendency (e.g. means) or other basic estimates (e.g. regression coefficient) AND variation (e.g. standard deviation) or associated estimates of uncertainty (e.g. confidence intervals) |
| ☒ | ☐ | For null hypothesis testing, the test statistic (e.g. *F*, *t*, *r*) with confidence intervals, effect sizes, degrees of freedom and *P* value noted<br>*Give P values as exact values whenever suitable.* |
| ☒ | ☐ | For Bayesian analysis, information on the choice of priors and Markov chain Monte Carlo settings |
| ☒ | ☐ | For hierarchical and complex designs, identification of the appropriate level for tests and full reporting of outcomes |
| ☒ | ☐ | Estimates of effect sizes (e.g. Cohen's *d*, Pearson's *r*), indicating how they were calculated |

*Our web collection on statistics for biologists contains articles on many of the points above.*

## Software and code

Policy information about availability of computer code

| | |
|---|---|
| Data collection | No software was used for data collection |
| Data analysis | Raw sequencing data was processed using CellRanger v2.0.1<br>scRNA-seq analysis was primarily performed using the R package "Seurat" v.4.3.1<br>R packages used include:<br>psupertime v0.2.6<br>fgsea v1.19.2<br>mgcv v1.8-38<br>liana 0.1.5<br>RcppML 0.5.6<br><br>Command line tools include:<br>Trimmomatic v0.36 |

For manuscripts utilizing custom algorithms or software that are central to the research but not yet described in published literature, software must be made available to editors and reviewers. We strongly encourage code deposition in a community repository (e.g. GitHub). See the Nature Portfolio guidelines for submitting code & software for further information.

## Data

Policy information about availability of data

All manuscripts must include a data availability statement. This statement should provide the following information, where applicable:

- Accession codes, unique identifiers, or web links for publicly available datasets
- A description of any restrictions on data availability
- For clinical datasets or third party data, please ensure that the statement adheres to our policy

Sixteen high-grade serous ovarian cancer datasets were obtained with permission from European Genome-Phenom Archive (EGAD00001006974). For kinase inhibitor-treated time-course experiment, raw sequencing files and processed UMI count matrices have been obtained from the NCBI Gene Expression Omnibus under the accession GSE147405. For OVCA420 time course treated with TGF-β1 experiment, raw sequencing files and processed UMI count matrices have been deposited in the NCBI Gene Expression Omnibus under the accession GSE247098.

## Research involving human participants, their data, or biological material

Policy information about studies with human participants or human data. See also policy information about sex, gender (identity/presentation), and sexual orientation and race, ethnicity and racism.

| | |
|---|---|
| Reporting on sex and gender | N/A |
| Reporting on race, ethnicity, or other socially relevant groupings | N/A |
| Population characteristics | N/A |
| Recruitment | N/A |
| Ethics oversight | N/A |

Note that full information on the approval of the study protocol must also be provided in the manuscript.

# Field-specific reporting

Please select the one below that is the best fit for your research. If you are not sure, read the appropriate sections before making your selection.

☒ Life sciences ☐ Behavioural & social sciences ☐ Ecological, evolutionary & environmental sciences

For a reference copy of the document with all sections, see nature.com/documents/nr-reporting-summary-flat.pdf

# Life sciences study design

All studies must disclose on these points even when the disclosure is negative.

| | |
|---|---|
| Sample size | Strict sample sizes were not selected a priori. As we sequenced the maximum amount of cells allowed per lane (approximately 10,000) in a 10x Genomics Chromium V2 for library generation, that would be the maximal sample size possible per sample in our OVCA420 experiment. |
| Data exclusions | Doublets were removed in downstream analysis. Possible dead or dying cells were also removed in downstream analysis by looking at expression of mitochondrial genes as a percentage of all genes |
| Replication | Replicate strategies are clearly stated in the manuscript. For time course data, a total of four replicates were performed, one per timepoint. internal controls were included in the design to ensure validity of the experiment. |
| Randomization | Randomization was largely not applicable for this study. |
| Blinding | Blinding was not relevant to this study. |

# Reporting for specific materials, systems and methods

We require information from authors about some types of materials, experimental systems and methods used in many studies. Here, indicate whether each material, system or method listed is relevant to your study. If you are not sure if a list item applies to your research, read the appropriate section before selecting a response.

## Materials & experimental systems

| n/a | Involved in the study |
| --- | --- |
| ☒ | ☐ Antibodies |
| ☐ | ☒ Eukaryotic cell lines |
| ☒ | ☐ Palaeontology and archaeology |
| ☒ | ☐ Animals and other organisms |
| ☒ | ☐ Clinical data |
| ☒ | ☐ Dual use research of concern |
| ☒ | ☐ Plants |

## Methods

| n/a | Involved in the study |
| --- | --- |
| ☒ | ☐ ChIP-seq |
| ☒ | ☐ Flow cytometry |
| ☒ | ☐ MRI-based neuroimaging |

# Eukaryotic cell lines

Policy information about cell lines and Sex and Gender in Research

| | |
| --- | --- |
| Cell line source(s) | OVCA420 cells come from a female with ovarian serous adenocarcinoma. OVCA420 cells were kindly provided by Dr. Gordon Mills (sourced originally from ascites of an ovarian cancer patient by Dr. Robert Knapp) |
| Authentication | For the OVCA420 cell line, 5e6 cells are lysed and undergo DNA extraction using a column based kit. Then the DNA is sent to TCAG.ca for DNA analysis. STR profiles were then checked against reference profiles. We have authenticated the OVCA420 cell line last in September of 2019. The experiment with OVCA420 cells was performed before that, in 2018. |
| Mycoplasma contamination | The cell lines tested negative for mycoplasma contamination. |
| Commonly misidentified lines (See ICLAC register) | None. |

# Plants

| | |
| --- | --- |
| Seed stocks | N/A |
| Novel plant genotypes | N/A |
| Authentication | N/A |
