# Supplementary material for: Mesenchymal ovarian cancer cells promote CD8+ T cell exhaustion through the LGALS3-LAG3 axis
Source: NPJ Syst Biol Appl. 2023 Dec 12;9:61. doi: 10.1038/s41540-023-00322-4 (PMC10716312; doi:10.1038/s41540-023-00322-4)
Supplement: Supplementary file 2 — Supplemental Figures [file 41540_2023_322_MOESM2_ESM.pdf]

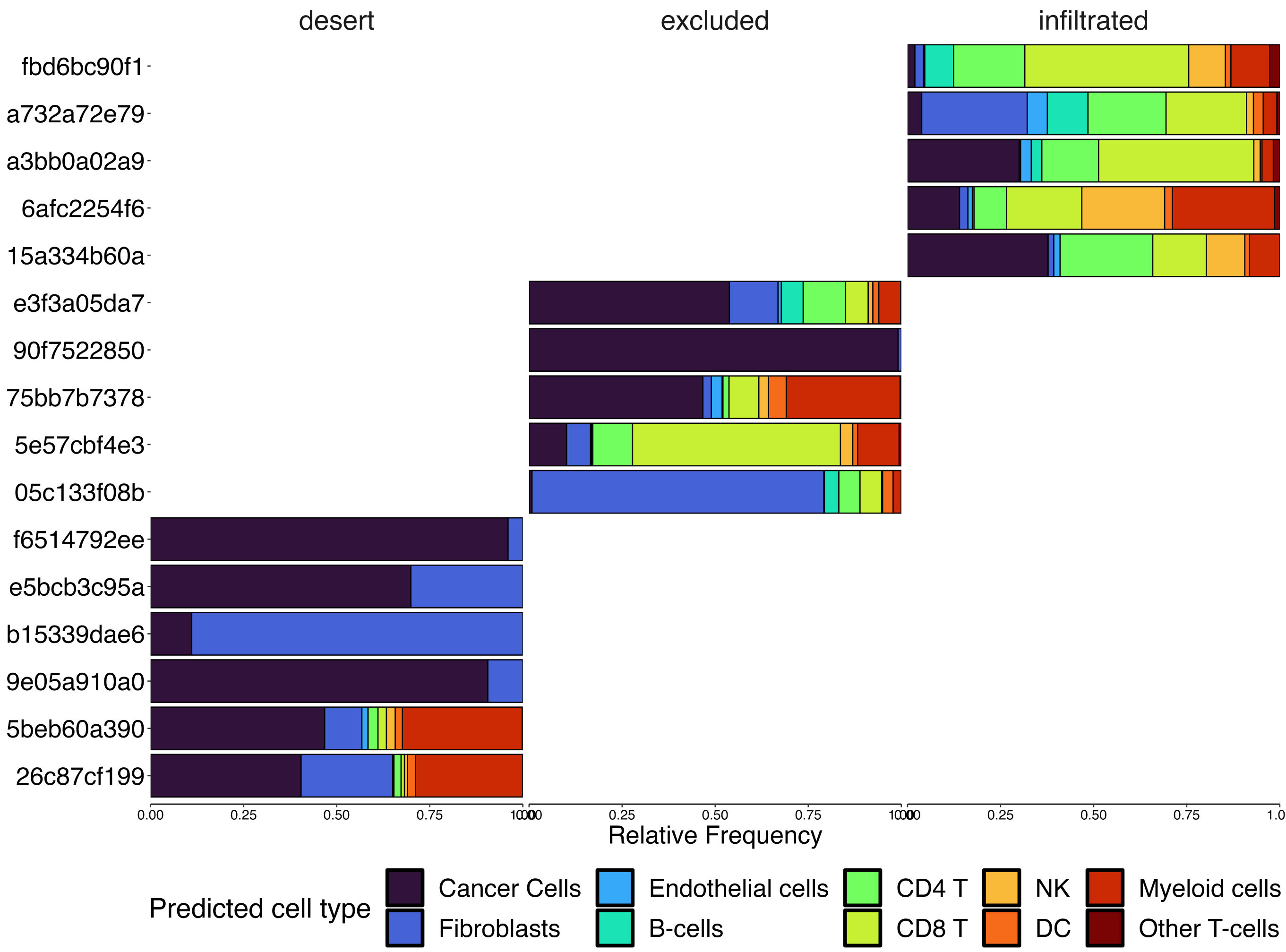

Supplemental Figure 1. **Individual infiltrated tumors have a larger proportion of immune cells compared to excluded or desert tumors.** Overall cell population proportions in patient derived tumor cells separated by patient ID and colored by cell population

a

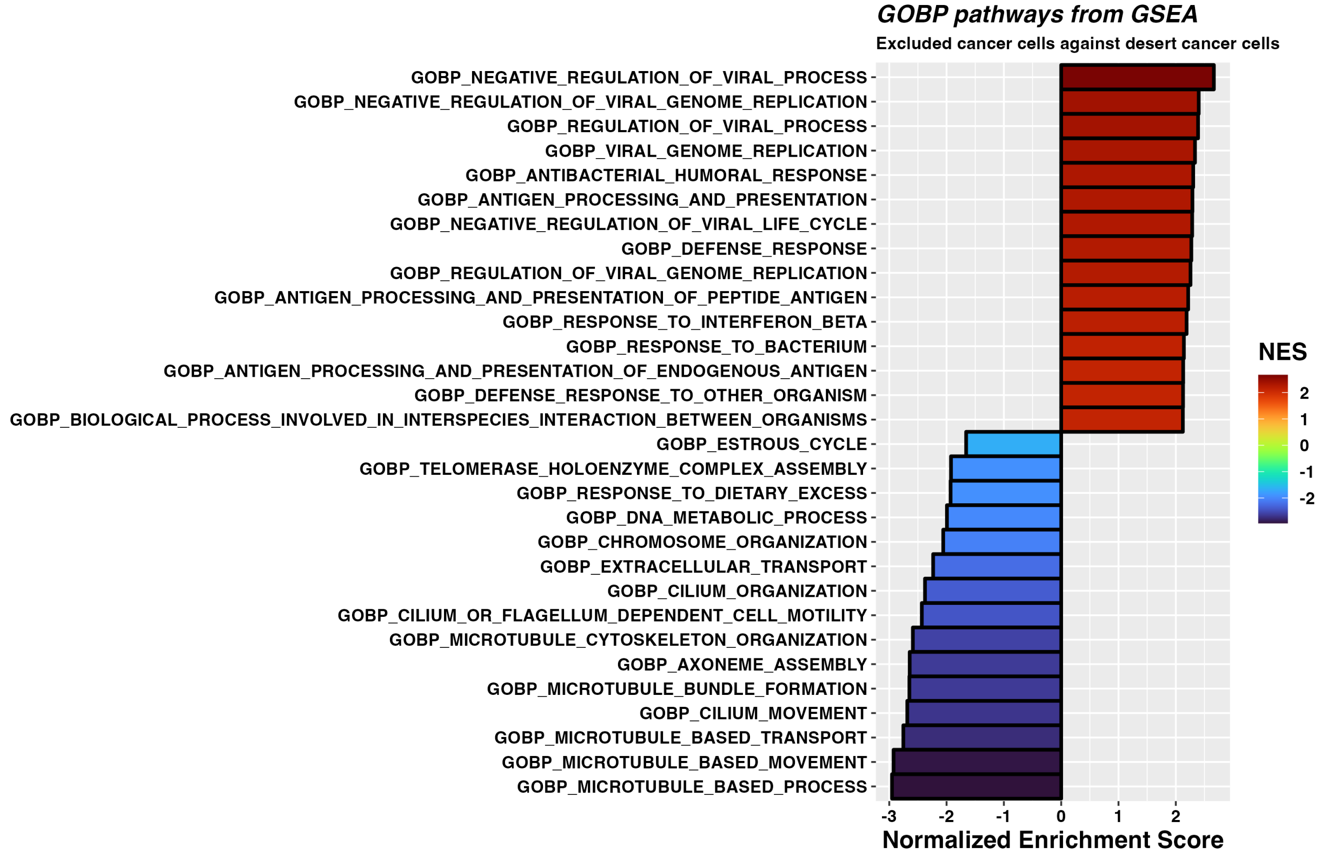

Supplemental Figure 2. **FGSEA analysis of enriched biological pathways in DEGs between cancer cells of infiltrated and excluded TMEs, and of excluded and desert TMEs.** FGSEA analysis of biological pathways enriched by DEGs overexpressed in cancer cells from excluded TMEs compared to cancer cells from desert TMEs

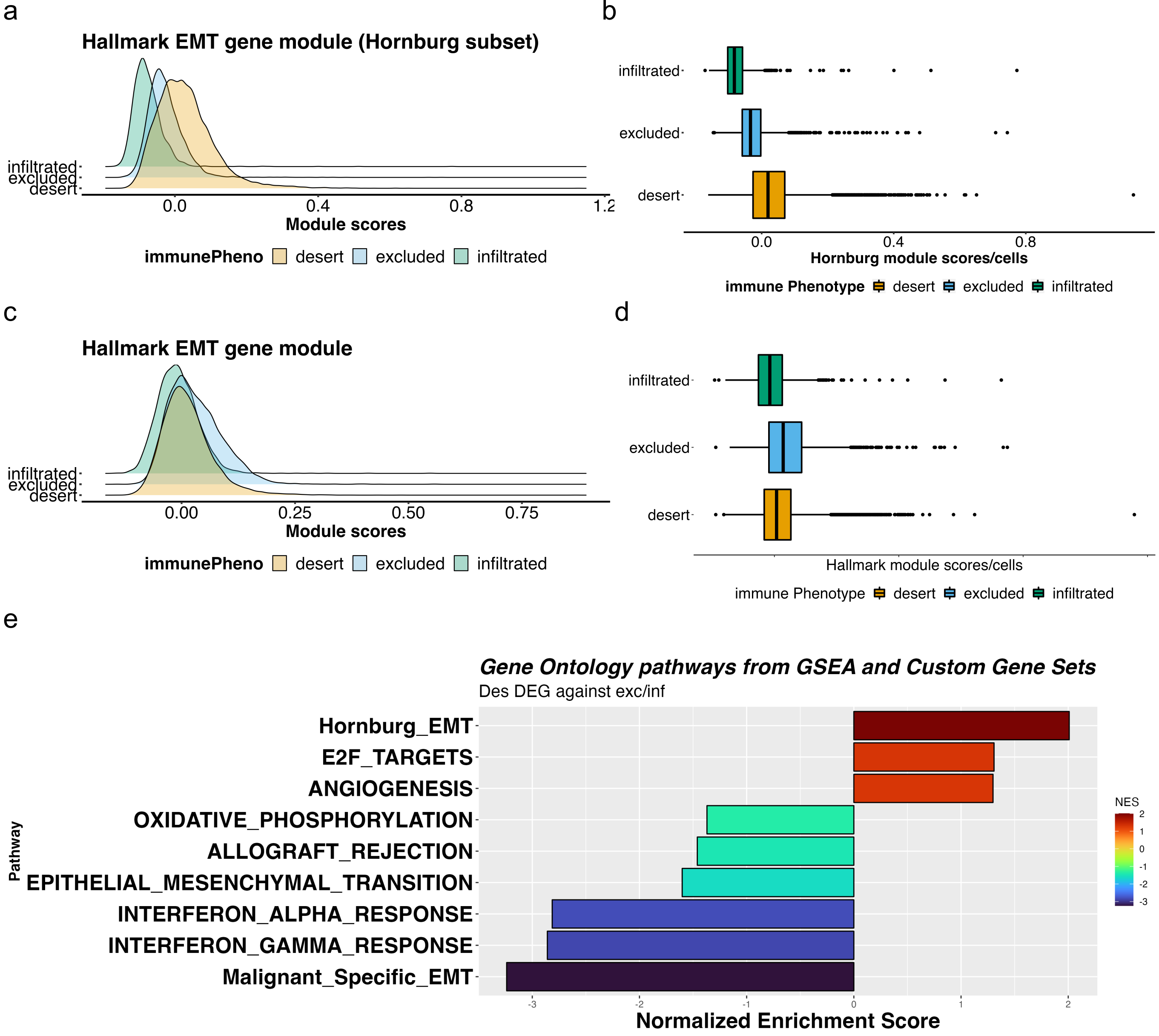

Supplemental Figure 3. **A comparison of different EMT gene module scores on cancer cells from TMEs with different immune phenotypes.** (a) Ridge plot of Hornburg EMT module scores (derivative of Hallmark EMT module) split by immune phenotypes (b) Boxplot of Hornburg EMT module scores split by immune phenotypes. (c) (Left) Ridge plot of Hallmark EMT module scores split by immune phenotypes (d) Boxplot of EMT Hallmark scores split by immune phenotypes. (e) GSEA enrichment analysis for three different EMT modules: select Hallmark EMT module from Hornburg et al., (2021) (Hornburg_EMT), our cancer-specific EMT module (Malignant_Specific_EMT), and Hallmark EMT module (EPITHELIAL_ MESENCHYMAL_TRANSITION) and other pathways either significantly enriched for in desert DEGs (red) or in excluded/infiltrated DEGs (green/blue).

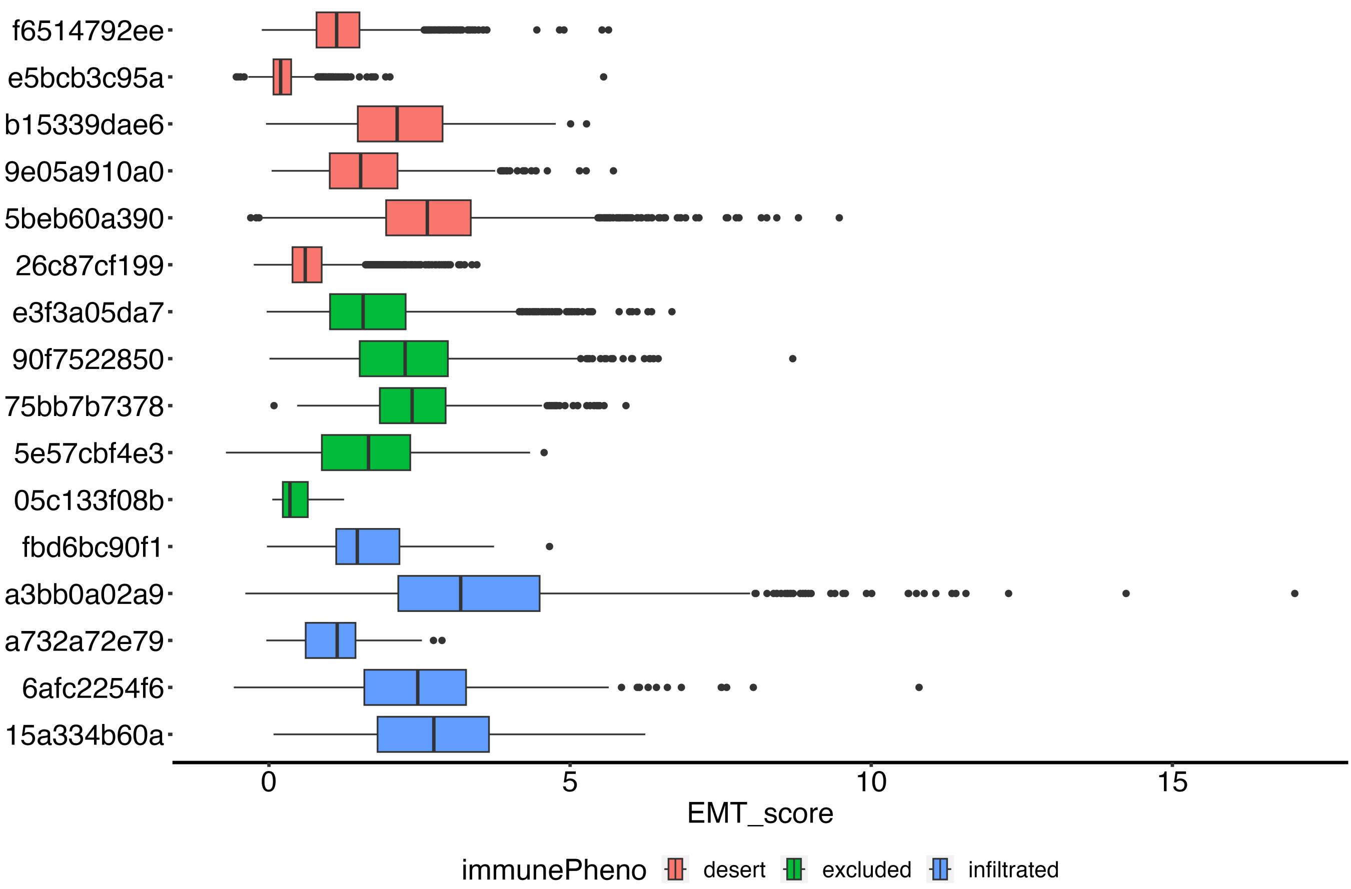

Supplemental Figure 4. **Individual infiltrated tumors contain cancer cells further along the EMT program compared to excluded or desert tumors.** Boxplot of cancer-specific EMT signature scores' dispersion in cancer cells arrayed by TME immune phenotype.

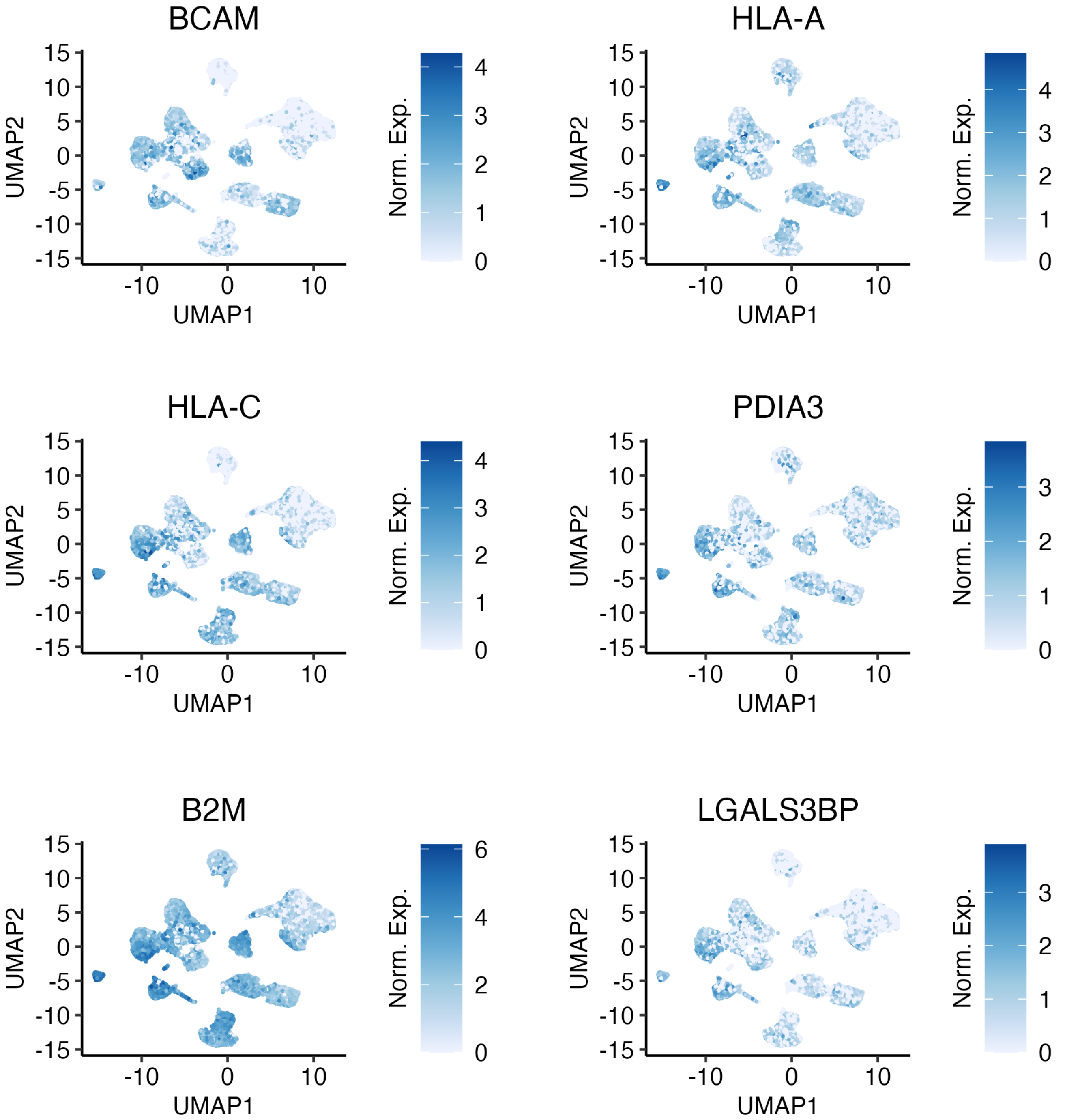

Supplemental Figure 5. **Cancer cells from infiltrated tumors have greater enrichment for select genes from the cancer-specific EMT module.** Enrichment UMAP plots for a subset of genes from the cancer-specific EMT signature module in the re-clustered cancer cell compartment.

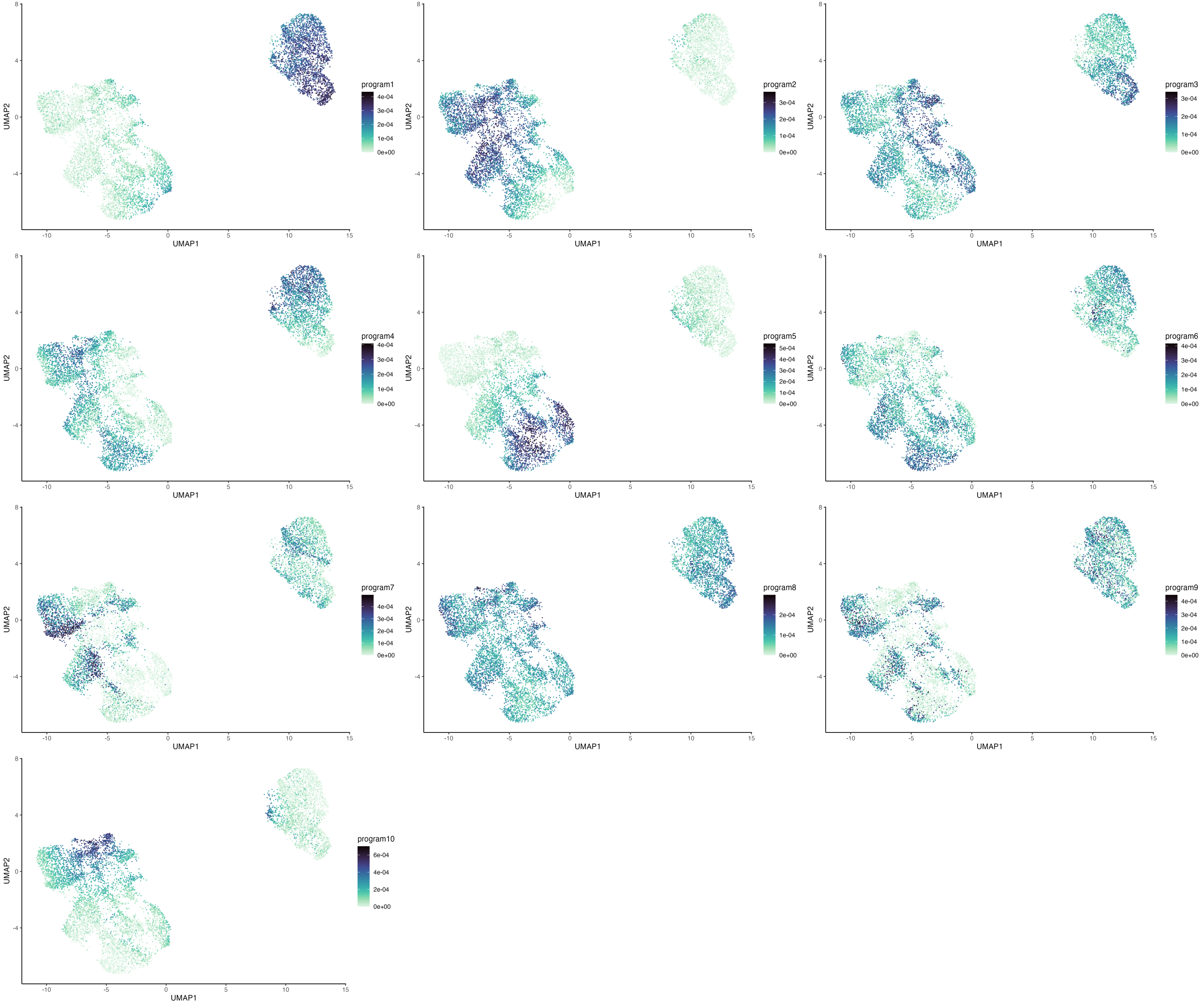

Supplemental Figure 6. **Enrichment of OVCA420 cells treated with TGF-β1 by 10 different programs generated through machine learning by application of NMF to the data**. All plots represent the ‘h’ coefficient of the NMF generated programs.

a

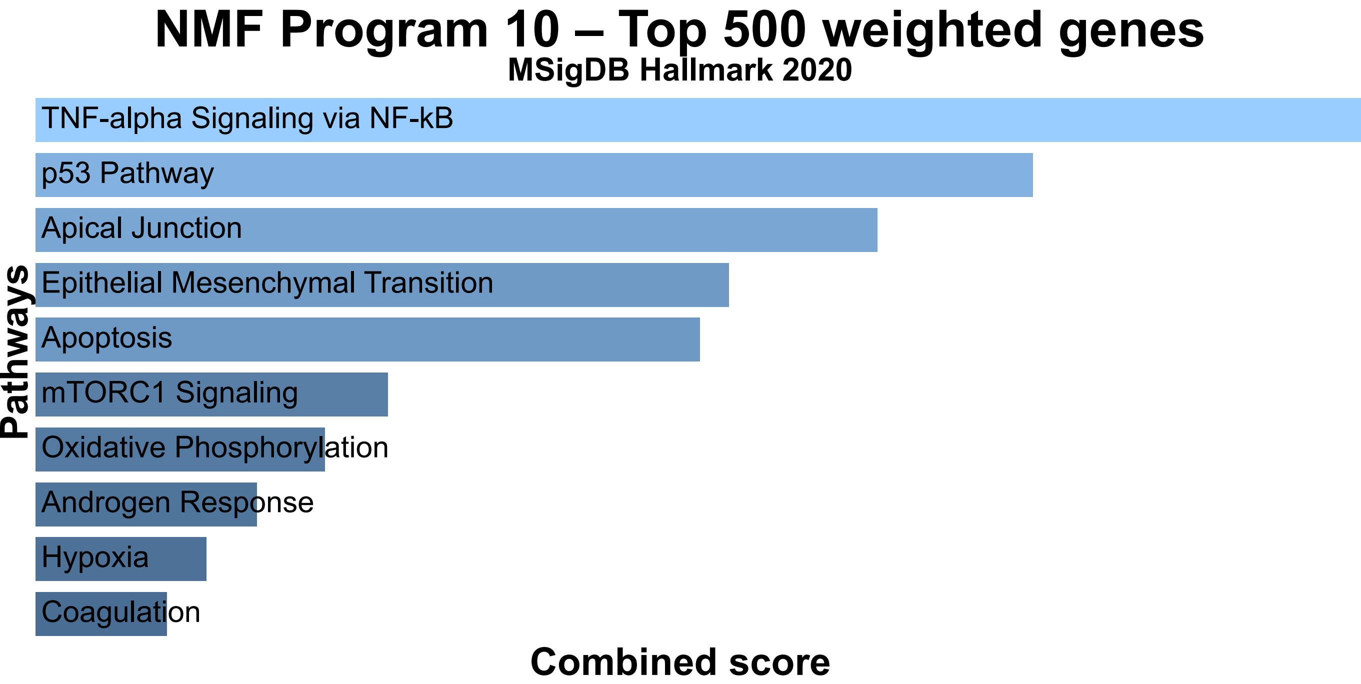

b

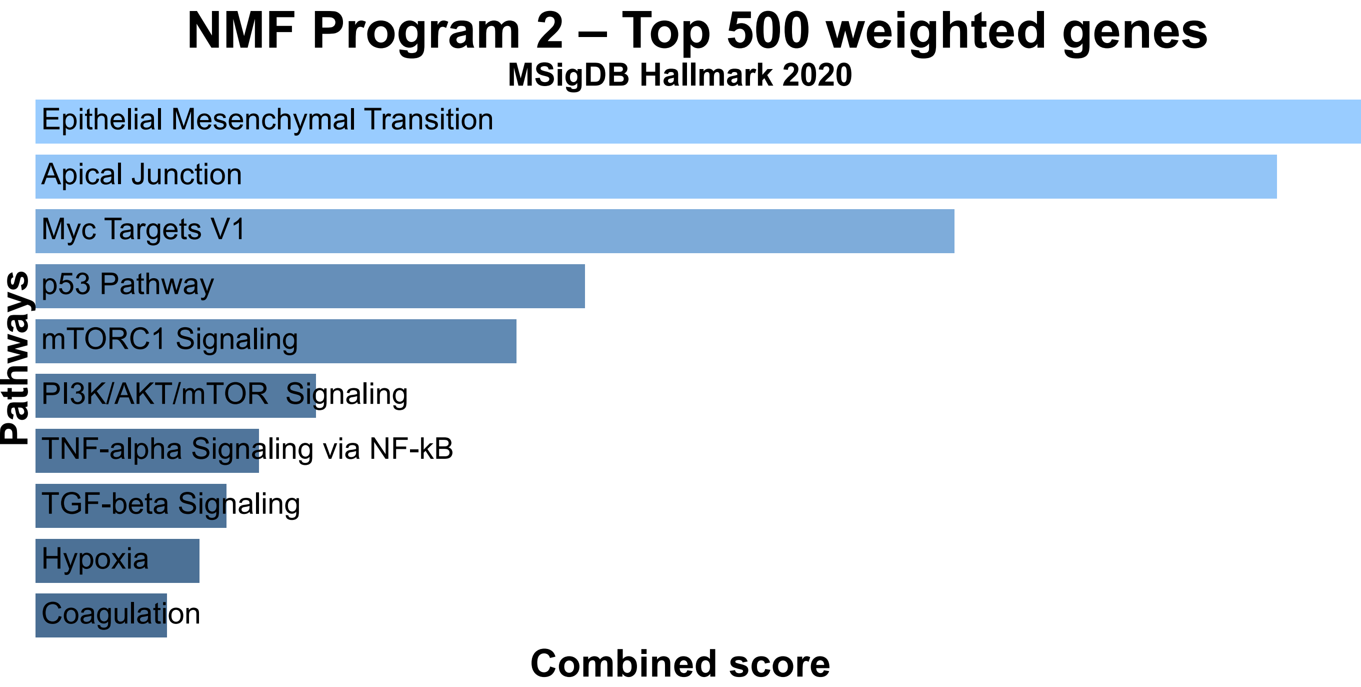

Supplemental Figure 7. **NMF Program 10 enriches for immunoregulatory signaling pathways.** (a) Enrichr analysis of top 500 genes, by weight, identified by NMF program 10 enriching for pathways in MSigDB Hallmark 2020 database. (b) Enrichr analysis of top 500 genes, by weight, identified by NMF program 2 enriching for pathways in MSigDB Hallmark 2020 database.

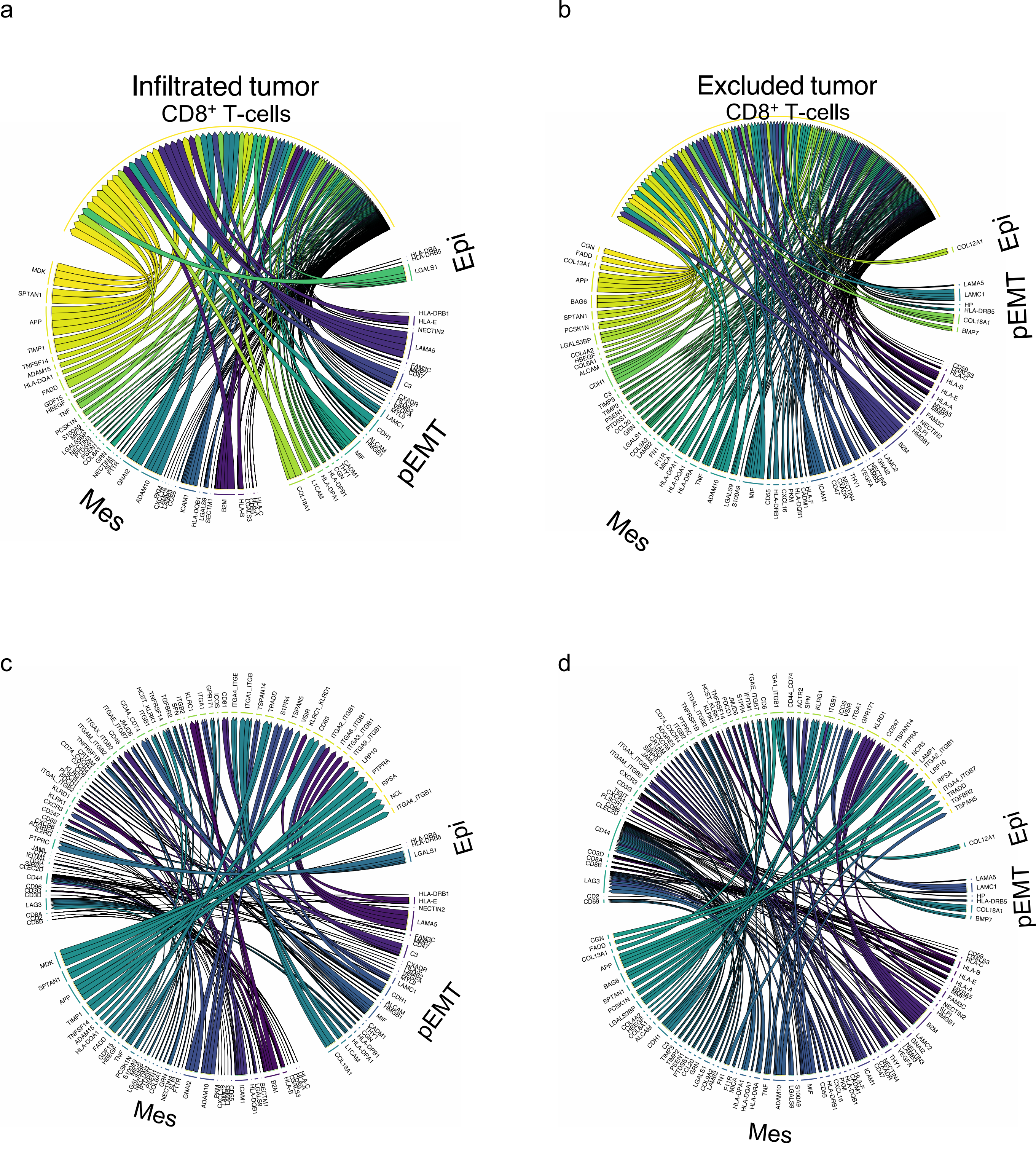

Supplemental Figure 8. **Most ligands targeting CD8⁺ T-cells in the TME originate from the mesenchymal cancer cells.** (a,b) Circle plot of ligands originating from cancer cells arrayed by delineated EMT status and interacting with recipient CD8⁺ T-cells in infiltrated (a) and excluded tumors (b). (c,d) Circle plot of ligands originating from cancer cells arrayed by delineated EMT status in infiltrated tumors (c) and excluded tumors (d), and the receptors targeted on recipient CD8⁺ T-cells.

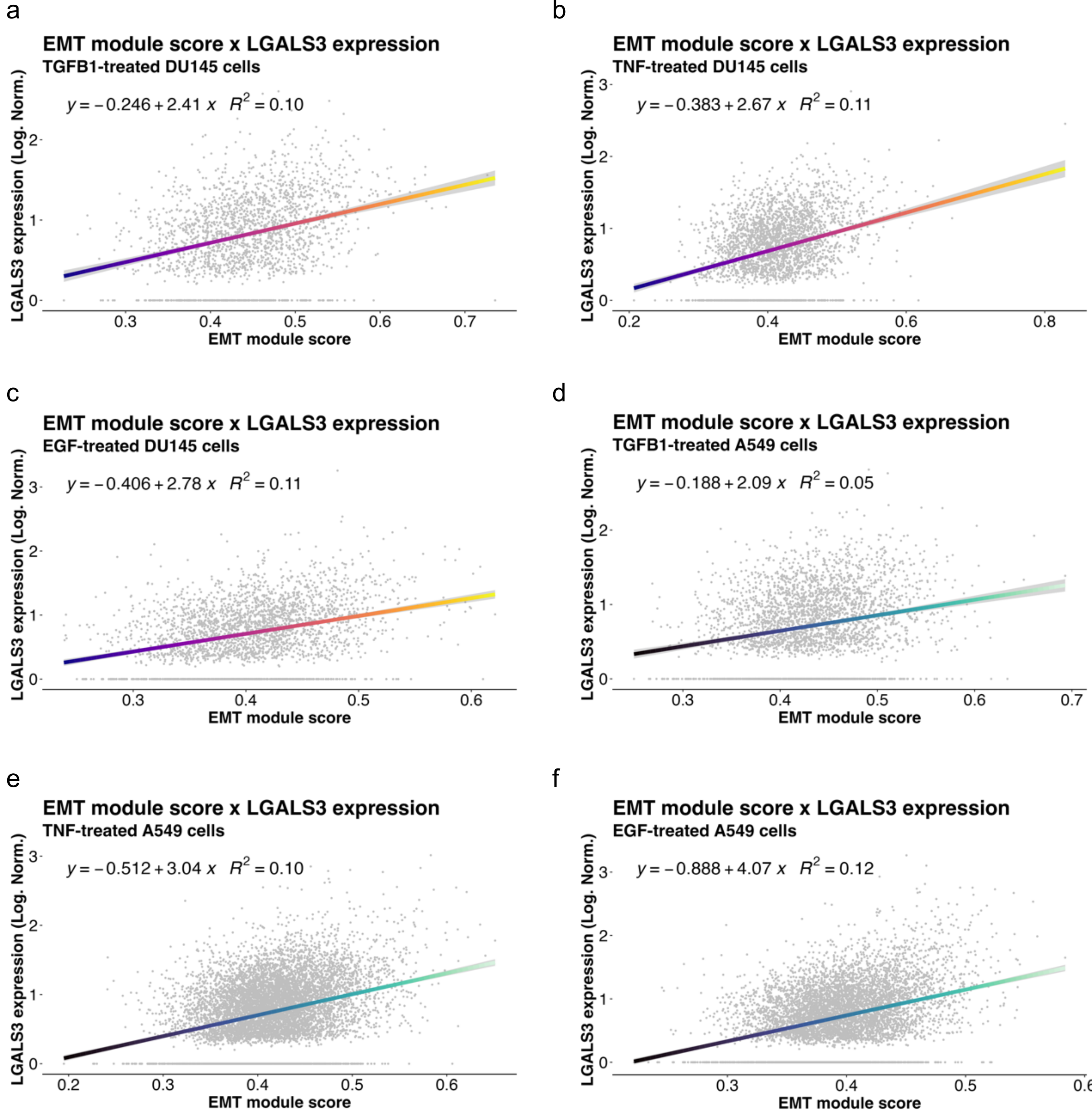

Supplemental Figure 9. **LGALS3 is directly correlated with the EMT *in vitro* in prostate and lung cancer cell lines.** (a-c) Log-normalized expression of LGALS3 GLM-correlated against cancer-specific EMT signature scores in DU145 prostate cancer cells treated with TGF-β1 (a), TNFα (b), and EGF (c). (d-f) Log-normalized expression of LGALS3 GLM-correlated against cancer-specific EMT signature scores in A549 lung cancer cells treated with TGF-β1 (d), TNFα (e), and EGF (f).

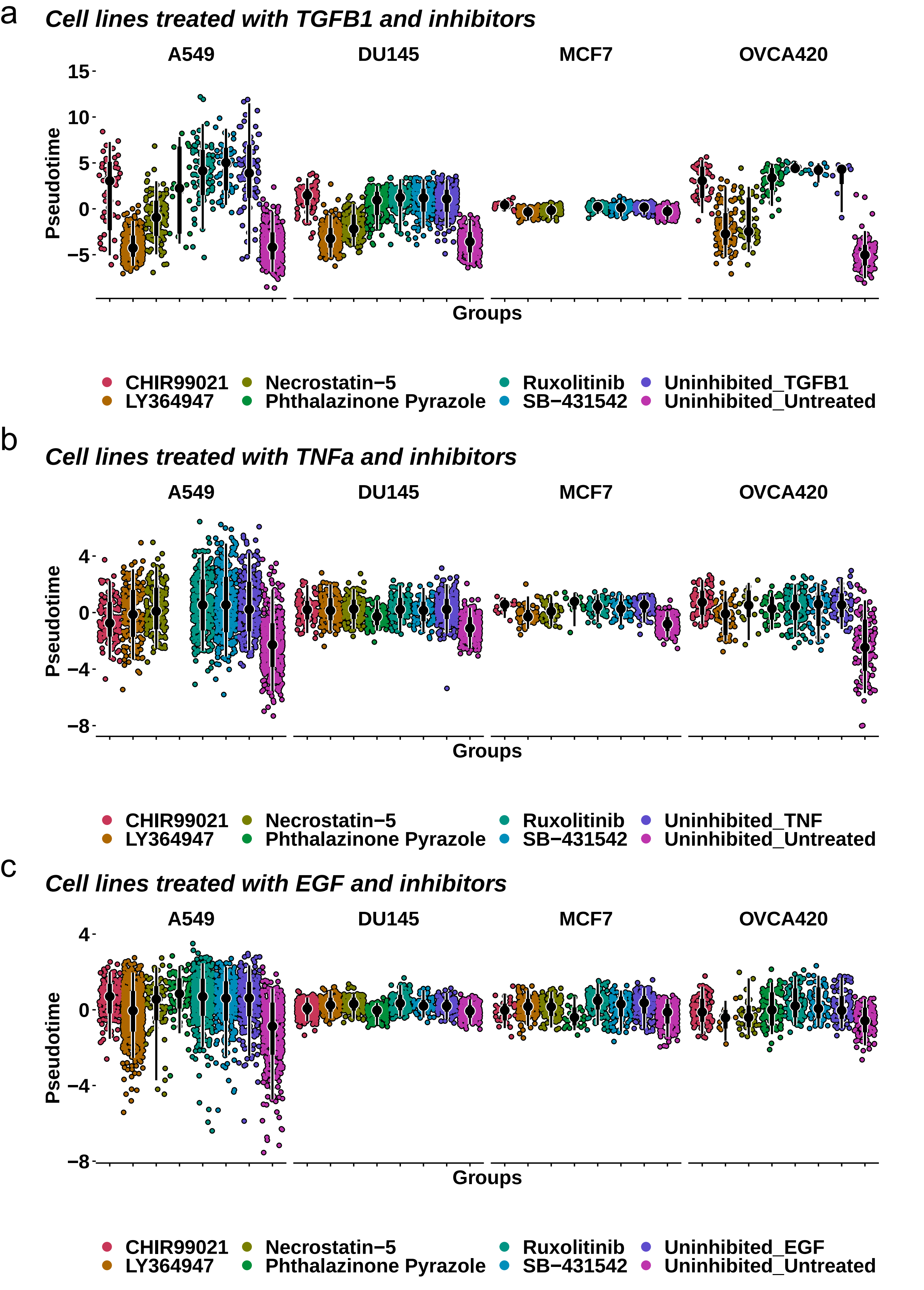

Supplemental Figure 10. **Pseudotime values across cell lines of different origins treated with different EMT inducers.** (a-c) Pseudotime values of A549, DU145, MCF7, or OVCA420 cell lines treated either with EMT inducers TGF-β1 (a), TNFα (b), EGF (c) or with EMT inducers and kinase inhibitors: RIP1 kinase inhibitor Necrostatin5, TGFβR1 kinase inhibitor LY364947, JAK1/2 kinase inhibitor Ruxolitinib, TGFβ/ALK kinase inhibitor SB-431542, GSK3 kinase inhibitor CHIR99021, or TGF-β1 and Aurora kinase A inhibitor phthalazinone pyrazole.
